# Determining Individual Variation in Growth and Its Implication for Life-History and Population Processes Using the Empirical Bayes Method

**DOI:** 10.1371/journal.pcbi.1003828

**Published:** 2014-09-11

**Authors:** Simone Vincenzi, Marc Mangel, Alain J. Crivelli, Stephan Munch, Hans J. Skaug

**Affiliations:** 1 Center for Stock Assessment Research, Department of Applied Mathematics and Statistics, University of California, Santa Cruz, Santa Cruz, California, United States of America; 2 Dipartimento di Elettronica, Informazione e Bioingegneria Politecnico di Milano, Milan, Italy; 3 Department of Biology, University of Bergen, Bergen, Norway; 4 Station Biologique de la Tour du Valat, Le Sambuc, Arles, France; 5 Fisheries Ecology Division, Southwest Fisheries Science Center, National Marine Fisheries Service, NOAA, Santa Cruz, Santa Cruz, California, United States of America; 6 Department of Mathematics, University of Bergen, Bergen, Norway; Princeton University, United States of America

## Abstract

The differences in demographic and life-history processes between organisms living in the same population have important consequences for ecological and evolutionary dynamics. Modern statistical and computational methods allow the investigation of individual and shared (among homogeneous groups) determinants of the observed variation in growth. We use an Empirical Bayes approach to estimate individual and shared variation in somatic growth using a von Bertalanffy growth model with random effects. To illustrate the power and generality of the method, we consider two populations of marble trout *Salmo marmoratus* living in Slovenian streams, where individually tagged fish have been sampled for more than 15 years. We use year-of-birth cohort, population density during the first year of life, and individual random effects as potential predictors of the von Bertalanffy growth function's parameters *k* (rate of growth) and 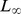 (asymptotic size). Our results showed that size ranks were largely maintained throughout marble trout lifetime in both populations. According to the Akaike Information Criterion (AIC), the best models showed different growth patterns for year-of-birth cohorts as well as the existence of substantial individual variation in growth trajectories after accounting for the cohort effect. For both populations, models including density during the first year of life showed that growth tended to decrease with increasing population density early in life. Model validation showed that predictions of individual growth trajectories using the random-effects model were more accurate than predictions based on mean size-at-age of fish.

## Author Summary

Somatic growth is a crucial determinant of ecological and evolutionary dynamics, since larger organisms often have higher survival and reproductive success. Size may be the result of intrinsic (i.e. genetic), environmental (temperature, food), and social (competition with conspecifics) factors and interaction between them. Knowing the contribution of intrinsic, environmental, and social factors will improve our understanding of individual population dynamics, help conservation and management of endangered species, and increase our ability to predict future growth trajectories of individuals and populations. The latter goal is also relevant for humans, since predicting future growth of newborns may help identify early pathologies that occur later in life. However, teasing apart the contribution of individual and environmental factors requires powerful and efficient statistical methods, as well as biological insights and the use of longitudinal data. We developed a novel statistical approach to estimate and separate the contribution of intrinsic and environmental factors to lifetime growth trajectories, and generate hypotheses concerning the life-history strategies of organisms. Using two fish populations as a case study, we show that our method predicts future growth of organisms with substantially greater accuracy than using historical information on growth at the population level, and help us identify year-class effects, probably associated with climatic vagaries, as the most important environmental determinant of growth.

## Introduction

A better understanding of growth will always be an important problem in biology. Somatic growth is one the most important life-history traits across taxa, since survival, sexual maturity, reproductive success, movement and migration are frequently related to growth and body size [1]. Variation in growth can thus have substantial consequences for both ecological and evolutionary dynamics [2]–[4].

Variation in growth can also affect the estimation of vital rates and demographic traits, which may translate to incorrect predictions of population dynamics [5]–[7]. However, the implications of including individual differences in growth in the study of population processes are largely unexplored, in part because of the computational challenges of estimating the determinants and the extent of shared (i.e. among homogeneous groups) and individual (i.e. after accounting for shared component) variation in growth [5], [8].

Determining how shared and individual variation in growth emerges may also improve predictions of future growth and size of individuals and populations, which is valuable for conservation and management [9], [10]. In addition, the ability to reliably predict missing body size data is crucial when testing for size-dependent survival and selection on body size. For instance, when estimating the effects of size on survival, the Cormack-Jolly-Seber model requires the size of individual to be known on the occasions when the individual was alive but not sampled, or, alternatively, it requires individual growth trajectories [11].

Thus, our understanding of growth dynamics and of its consequence on population and evolutionary dynamics can greatly benefit from the use of new computational approaches that are able to tease apart the sources of growth variation. The accurate estimation of parameters of growth models is particularly useful in this regard, since it reduces the information provided by a potentially long series of measurements to a few values that summarize the most relevant process governing growth and can then be used to tease apart individual and shared determinants of growth variation [12].

Multiple processes contribute to the realized growth of organisms, such as individual variation, size-selective mortality, annual and spatial variation in growth, intra- and inter-specific competition. Understanding the nature and contribution of these multiple sources of variation in growth faces a number of methodological challenges. First, to simultaneously estimate shared and individual contributions to the observed variation in growth require longitudinal data. In fact, when data are cross-sectional, it is rarely possible to separate variation in growth that emerges from persistent differences among individuals from variation due to stochastic processes [13]. Second, especially for mobile organisms, it is seldom possible to obtain more than a few observations for an individual throughout its lifetime (i.e. temporal data are often sparse). Thus, data for a particular individual are unlikely to be adequate for the estimation of parameters of the growth model for that individual and additional information may be needed, such as data of other individuals thought to be similar (i.e. “borrowing strength” or “shrinkage” [14]). Models in which the estimate of each effect is influenced by all members in a group are alternatively called hierarchical, random-effects, multilevel, or mixed models [14]. For consistency, in this paper we only use the term random-effects model. Modeling and estimating random effects also have the advantage of addressing the lack of independence between repeated measurements of the same individuals and of individuals in homogeneous groups [15]. In addition, when using parametric growth models, parameter estimates at the individual or shared level as well as their correlation structure can lead to insights on the processes governing the growth of individuals or group of individuals. Third, since no organism can growth without bound, growth models must at some point be non-linear [16], [17] and the estimation of model parameters is thus computationally demanding. Generally, fast and reliable approaches are needed in order to investigate multiple parameterizations of growth models. In this work, we propose a stable, reliable, and fast parametric Empirical Bayes (EB) approach [18]–[20] for estimating shared and individual variation in somatic growth using longitudinal data and random-effects models [8], [10], [21].

To illustrate the power and generality of our methods, we consider long-term studies of two populations of marble trout *Salmo marmoratus* living in Slovenian streams. Both populations have been sampled annually for more than 15 years, and show substantial differences within and among populations in the mean growth of cohorts and in size-at-age of individuals. In [22], we demonstrated that fast-growing marble trout allow population recovery after massive mortality events, such as those caused by floods and landslides, due to the positive influence of larger size-at-age of fish on recruitment. In addition, because observed variation in growth among individuals is heritable [23], there is potential for the evolution of growth rates toward faster growth in populations affected by massive mortality events [24], [25].

However, how variation in growth in marble trout is determined by shared and individual factors is unknown. Within populations of the same species, persistent differences in growth are commonly observed both among groups (e.g. year-of-birth cohorts, families) and among individuals within groups. Cohort effects are often induced early in life and have the potential to strongly affect the performance of individuals throughout their lifetime [26]–[29]. These early effects on lifetime growth may reflect either constraints or adaptations [29], and are often ascribed to climatic vagaries during early development that similarly affect the whole cohort [30]–[32]. In marble trout as well as in other species, population density during the early life stages also has substantial effects on lifetime growth, in particular due a reduction in per-capita food availability [33] or the occupation of spaces of low profitability with increasing population density [34], [35]. At the population level or within more homogenous groups, among-individual variation may emerge from differences in overall genetic growth potential, metabolic rates, behavioral traits (e.g. aggressiveness), occupation of sites of different profitability, or life-history strategies (e.g. partition of energy to competing functions, such as growth, storage, reproduction and maintenance) [36].

In this paper, we show how new computational methods for the estimation of a parameter-rich non-linear growth function using longitudinal data can shed light on the shared and individual determinants of somatic growth in natural populations. Our aim is to expand the toolkit available to biologists rather than proposing a method globally superior to another, since the particular biological problem should play an important role in selecting the tool. The paper is organized as follows. First, we introduce the two populations of marble trout that we used as a motivating example and case study. We then present the Empirical Bayes approach to parameter estimation as implemented in the module ADMB-RE (Automatic Differentiation Model Builder - Random-Effects) of the software ADMB [37] and apply the EB approach to the joint estimation of shared and individual variation in growth from longitudinal data using a parameter-rich von Bertalanffy growth function. For the case study of marble trout, we introduce environmental predictors of the von Bertalanffy growth function's parameters *k* (rate of growth) and 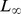 (asymptotic size) such as population density in the first year of fish life and year-of-birth cohort, and test whether their inclusion, in addition to individual random effects, improves model performance. We compare the parameter estimates and resulting estimated growth trajectories for two populations of marble trout living in different habitats and showing different demographic traits, and highlight shared and contrasting results between the two populations. We then test the ability of the growth model to predict unobserved length-at-age of individual fish. We discuss the life-history mechanisms that may generate the observed patterns of growth, as well as their implications for population dynamics.

## Materials and Methods

### Ethics statement

All sampling work was approved by the Ministry of Agriculture, Forestry and Food of Republic of Slovenia and the Fisheries Research Institute of Slovenia. Original title of the Plan: RIBISKO - GOJITVENI NACRT za TOLMINSKI RIBISKI OKOLIS, razen Soce s pritoki od izvira do mosta v Cezsoco in Krnskega jezera, za obdobje 2006–2011. Sampling was supervised by the Tolmin Angling Association (Slovenia).

### Case study

Our case study involves two populations (Gacnik and Zakojska) of marble trout living in Slovenian streams [38]. These populations are part of a larger study involving 10 marble trout populations [38]. We limit our discussion to two populations showing contrasting demographic traits and characteristics of the habitat to focus on the computational tools and the insights generated by the estimation of the parameters of the growth models.

Marble trout is a resident salmonid endemic in Northern Italy and Slovenia that is now endangered due to widespread hybridization with introduced brown trout and displacement by alien rainbow trout. The populations of Gacnik and Zakojska were established in stretches of fishless streams in 1996 (Zakojska) and 1998 (Gacnik) by stocking age-1 fish that were the progeny of parents from relic genetically pure marble trout populations [39]. Trout in Gacnik and Zakojska are genetically different [39]. Fish hatched in the streams for the first time in 1998 and in 2000 in Zakojska and Gacnik, respectively. Those cohorts are the first included in the analysis. The two populations were sampled annually in June. The geomorphological characteristics of the two streams are different: Zakojska is (mostly) a fragmented one-way stream (i.e. trout can move downstream, but not upstream), while Gacnik is a two-way stream, i.e. trout can move in either direction.

**Figure 1:**
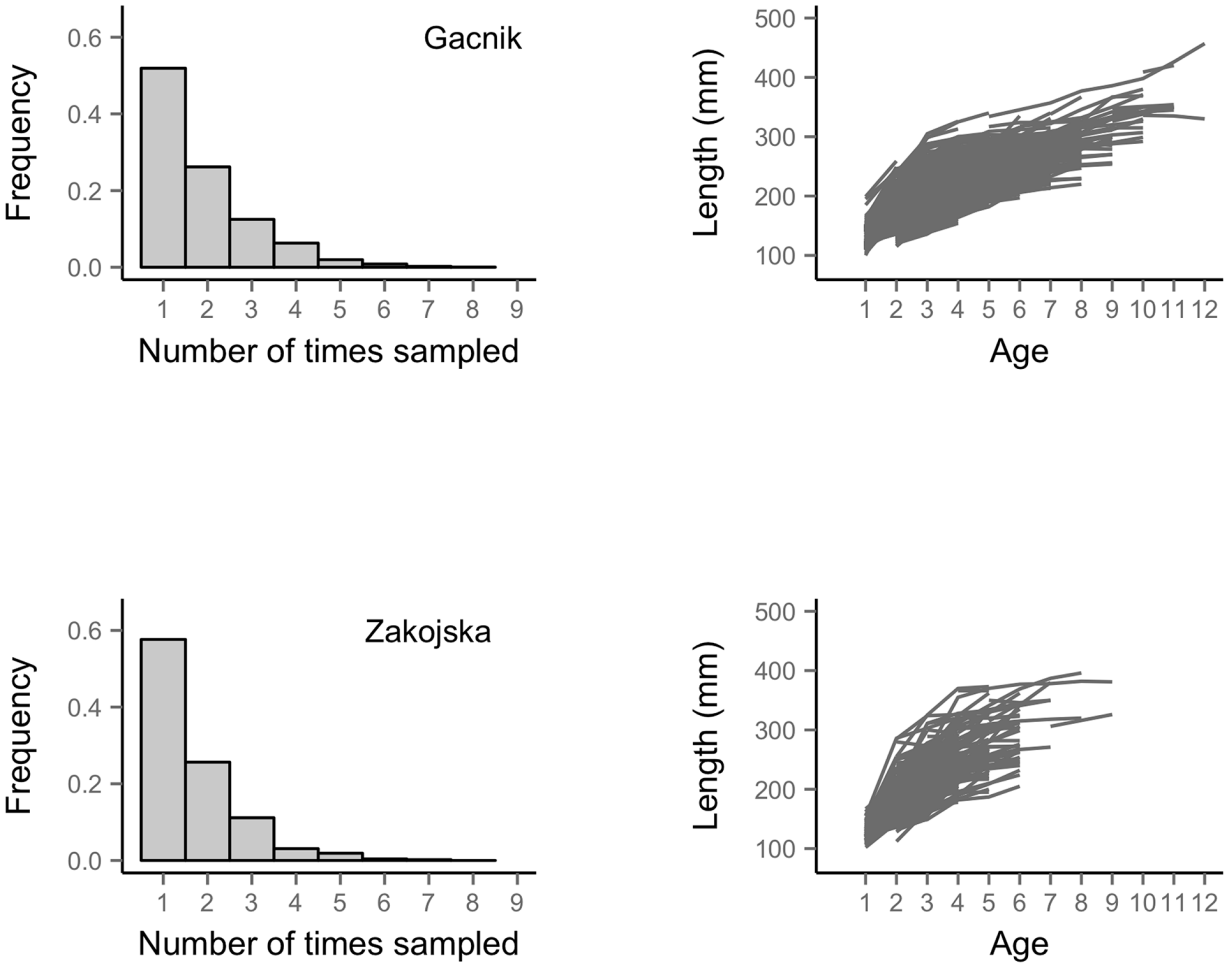
Number of recaptures for fish and observed growth trajectories. Frequency of number of capture/recaptures for fish (left column) and observed individual growth trajectories (right column) for the populations of Gacnik (top row) and Zakojska (bottom row).

Fish were collected by electrofishing and measured for length and weight to the nearest mm and g, respectively (fig. 1). If fish were caught for the first time - or if the tag had been lost – and they were longer than 110 mm they were tagged with Carlin tags [40] and age was determined by reading scales. Marble trout spawn in November–December and offspring emerge in April–May. Underyearlings are smaller than 110 mm in June, thus trout were tagged at age 1 or, in the case of small size, at age 2. Males and females are morphologically indistinguishable at the time of sampling. The probability of recapture was higher than 80% and we did not find evidence of capture probability varying with age and size for fish older than age 0 [41]. In addition, we found no evidence of size-selective mortality in either stream [42]. The movement of marble trout is limited, and the majority of marble trout were sampled within the same 200 m reach throughout their lifetime. Marble trout females achieve sexual maturity when longer than 200 mm, usually at age 3 or older. The maximum observed age for fish born in the streams was 12 and 9 years in Gacnik and Zakojska, respectively. The last sampling occasion included in the dataset was June 2012. In Gacnik the last cohort included was the one born in 2010. Due to a flood that almost completely wiped out the population in 2007 [38], the last cohort included for Zakojska was the one born in 2008. Density of fish of age 1 and older (number m^−2^) was (mean±sd) 0.05±0.04 in Zakojska from 1998 to 2012 and 0.16±0.07 in Gacnik from 2000 to 2012. In total, 1 067 unique fish were included in the Zakojska dataset and 4 764 in the Gacnik dataset.

### Empirical Bayes method for random-effects models

Empirical Bayes (EB) refers to a tradition in statistics where the fixed effects and variance (or standard deviation) of a random-effects model are estimated by maximum likelihood, while estimates of random effects are based on Bayes formula (e.g. [43], [44]). Although random-effects models can be analyzed using frequentist or Bayesian methods [45], [46], the frequentist point of view may have a number of advantages [44]. From a computational perspective the maximum likelihood estimate is relatively inexpensive to calculate and avoids difficulties associated with judging convergence of MCMC samplers when using a full Bayesian approach. Due to these advantages, EB has increasingly been applied in the last few years in the biological sciences in fields including genetics [47], [48], disease screening [49], and genomics [50]. Estimation of fixed effects and variance parameters by maximum likelihood is in widespread use in mixed model software packages, such as the R package “lme4” [51].

#### Mathematical notation

We denote fixed effects by Greek letters (*α*,*β*,…), with 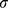 being reserved for standard deviations; roman letters (*u*,*v*,…) are used for the random effects. We describe a general three level model with individuals (indexed by 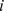) being nested within groups (index by 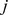), which again are nested within population. For computational reasons we only allow random effects to occur at the individual level, although in principle a full hierarchy of effects is possible. There exist several general guidelines about which parameters in a hierarchical model should be taken as random effects [14], [52]; our considerations are mostly pragmatic, aimed at both obtaining parameter estimates instrumental for understanding the system investigated and facilitating rapid and stable estimation of parameters.

As a general example, we use a standard Generalized Linear Mixed Model (GLMM) [15] to link the model parameters to fixed and random effects, as well as to present the parameter estimation algorithm. That is, consider a parameter *g* of a process (e.g., growth, survival, movement) for an individual *i* in group *j* (e.g. cohort) as a function of a continuous predictor 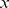 (which may be specific to individual *i* in group *j*), e.g. density of individuals. The linear predictor of the process parameter *g* is
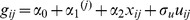
(1)where 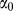 is the intercept, 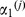 is the group effect, 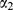 is a regression parameter on the continuous variable *x_ij_*, *u_ij_* ∼*N*(0,1) are standardized individual random effects and *σ_u_* is the standard deviation of the statistical distribution of the random effects. The covariate term *α*_2_ *x_ij_* also yields individual variation, but of a different type than that provided by the random effect.

When *g* is the parameter of a dynamic process (e.g. growth), the random effect term 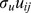 induces correlation among observations made on the same individual, and 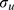 in effect becomes a correlation parameter. Thus, the parameters to be estimated by maximum likelihood are 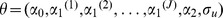 (i.e. the population parameters), where *J* is the number of groups (e.g. year-of-birth cohorts).

If *f*(data,*u_ij_*;***θ***) denotes the joint probability density of the data and random effect for a given individual, we obtain the marginal likelihood function by integration
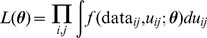
(2)This formulation is computationally attractive because the likelihood is a product over one-dimensional integrals. Had we taken the 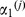 as random effects, the need to integrate also over 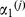 would have yielded a joint integral over 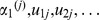. In presence of other parameters with a potential individual component (i.e. *g*_1_, *g*_2_ etc.), each *g* has its own version of the linear predictor (with random effects 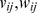 etc.). However, the marginal likelihood in Eq. 2 is still computationally efficient, since each integral is taken over a small number of random effects 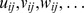.

The full suite of frequentist inference tools is valid for inference about the population parameters (elements of ***θ***). As described above, for the random effects the EB method applies Bayesian principles. Given a maximum likelihood estimate of ***θ***, the estimate of *u_ij_* is the mean or mode of the posterior distribution. The latter (used in ADMB-RE) is obtained by maximizing the integrand *f*(data*_ij_*,*u_ij_*;***θ***) in Eq. 2 with respect to 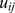, with ***θ*** fixed at its maximum likelihood estimate. The “borrowing strength” aspect of EB is that the standard normal prior placed on 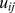 “pulls” its estimate toward zero (the population value) to an extent that depends on 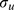, which is estimated from the full dataset.

### Fitting non-linear random-effects models in ADMB-RE

ADMB is an open source statistical software package for fitting non-linear statistical models [37], [53]. ADMB can be used to fit generic random-effects models with an EB approach using the Laplace approximation (ADMB-RE [54]). ADMB is totally flexible in model formulation, allowing any likelihood function to be coded in C++. Coding in C++ allows also for a great flexibility of functional forms to be used for model parameterization. In terms of computing times, ADMB compares favorably to other software and methods for the estimation of parameters of highly-complex non-linear models [55] (see also text S2).

The gradient (i.e. the vector of partial derivatives of the likelihood function with respect to model parameters) provides a measure of convergence of the parameter estimation procedure in ADMB. Although considerations of speed and model complexity may motivate the use of a less strict convergence criterion, by default ADMB stops when the maximum gradient component (i.e. the largest of the partial derivatives of the likelihood function with respect to model parameters) is <10^−4^. An explicit convergence criterion allows the researcher to systematically move forward in the analysis and thus potentially explore a large number of model parameterizations.

### Growth model

A broad range of models describing the physiology of growth have been developed [56]–[62] and recent papers have summarized non-linear growth models along with methods for parameter estimation [16], [17]. However, it has often been difficult, if not impossible, to estimate parameters for many of the proposed growth models using data on individual growth trajectories in natural settings. Even in the presence of a large amount of data, a highly parameterized model may be only weakly statistically identifiable.

We use the growth model due to von Bertalanffy [59], [63], [64]. The von Bertalanffy growth function (vBGF) has been used to model the growth of organisms across a wide range of taxa, including fish [57], [65], mammals [66], [67], snakes [68], and birds [69], [70]. von Bertalanffy hypothesized that the growth of an organism results from a dynamic balance between anabolic and catabolic processes [59]. If *W*(*t*) denotes mass at time *t*, the von Bertalanffy assumption is that anabolic factors are proportional to surface area, which scales as 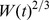, and that catabolic factors are proportional to mass. If *a* and *b* denote these scaling parameters, then the rate of change of mass is
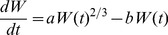
(3)If we further assume that mass and length, *L*(*t*), are related by 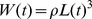 with *ρ* corresponding to density, then elementary calculus shows that [64]

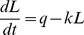
(4)where 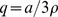 and 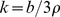.

The linear differential equation in Eq. 4 is readily solved by the method of the integrating factor. Setting 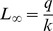 to be the asymptotic size (obtained when we set the left-hand side of Eq. 4 equal to 0) and 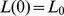 to be the initial size, two forms of the solution are
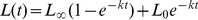
(5)and
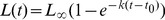
(6)where *t*_0_ is the hypothetical age at which length is equal to 0.

In light of Eq. 4, if 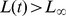, the rate of growth is negative, so that we can think of asymptotic size as the size only attained in the limit of very long times. For a given value of asymptotic size, the parameter *k* (in y^−1^) describes how fast the individual or group of individuals reaches the asymptotic size. In this work, we will use the formulation of the vBGF of Eq. 6, which has 3 parameters: *L*_∞_, *k*, and *t*_0_. Although the mechanistic definition of asymptotic size in the vBGF introduces an explicit linear relationship on the log scale between *k* and *L*_∞_ (i.e. 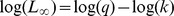), in this work we do not explicitly introduce *L*_∞_ as equal to 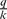, but we let the correlation between *L*_∞_ and *k* at the whole population and at the individual level emerge from data, as it is commonly done [71].

#### Parameter estimation and individual variation

In the vast majority of applications of the vBGF, *L*_∞_, *k*, and *t*_0_ have been estimated at the population level (i.e. without accounting for individual heterogeneity in growth) starting from cross-sectional data, and interpreted as the growth parameters of an average individual in the population (e.g. *L*_∞_ is the asymptotic size of an average individual). That is, one collects a group of individuals at a single time, measures their sizes and ages, and then estimates the parameters of the vBGF growth function parameters at the population (or groups within populations, e.g. cohorts) level using standard non-linear regression techniques via maximum likelihood or Bayesian methods ([72] and references therein). However, when data include measurements on individuals that have been sampled multiple times, failing to account for individual variation in growth will lead to biased estimations of mean length-at-age [13], [73].

Following [58] (although in [58] data were not longitudinal and thus individual random effects were not included), we present a formulation of the vBGF specific for longitudinal data where *L*_∞_, *k*, and *t*_0_ may be allowed to be a function of shared predictors and individual random effects. To improve the biological interpretation of the parameters of the vBGF, we treated *t*_0_ in Eq. 6 as a population-level parameter (with no predictors), so that all individuals are assumed to have a shared value. Since *k* and *L*_∞_ must be non-negative, it is natural to use a log-link function. In addition, values far apart on the natural scale are often of the same magnitude when log-transformed; this facilitates parameter estimation and model convergence. We thus set
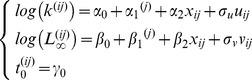
(7)where 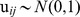 and 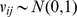 are the standardized individual random effects, 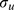 and 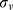 are the standard deviations of the statistical distributions of the random effects, and the other parameters are defined as in Eq. 1. The minimal model (i.e. with no predictors and 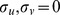) only contains 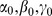. The continuous predictor *x_ij_* in Eq. 7 (i.e. population density in our analyses, but one may have different continuous predictors for *L*_∞_ and *k*) does not need to enter linearly into the equation, i.e. any of the terms 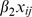 and 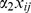 may be replaced by a more general function *h*(*x_ij_*;*Φ*), where *Φ* denotes a set of parameters to be estimated.

We thus assume that the observed length of individual *i* in group *j* at age *t* is
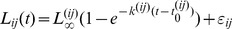
(8)where *ε_ij_* is normally distributed with mean 0 and variance 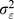.

To focus on the EB method, we do not explicitly introduce process stochasticity, so that the likelihood function is
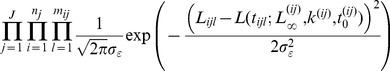
(9)where *n_j_* is the number of individuals in group *j*, *m_ij_* is the number of observations from individual *i* of group *j*, *l* is an index that runs over these observations, the observed length measurements for individual *i* in group *j* are denoted by 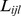, while 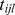 is the age of the individual when the *l*-th measurement is made. Predictors are implicitly included via Eq. 8.

**Figure 2:**
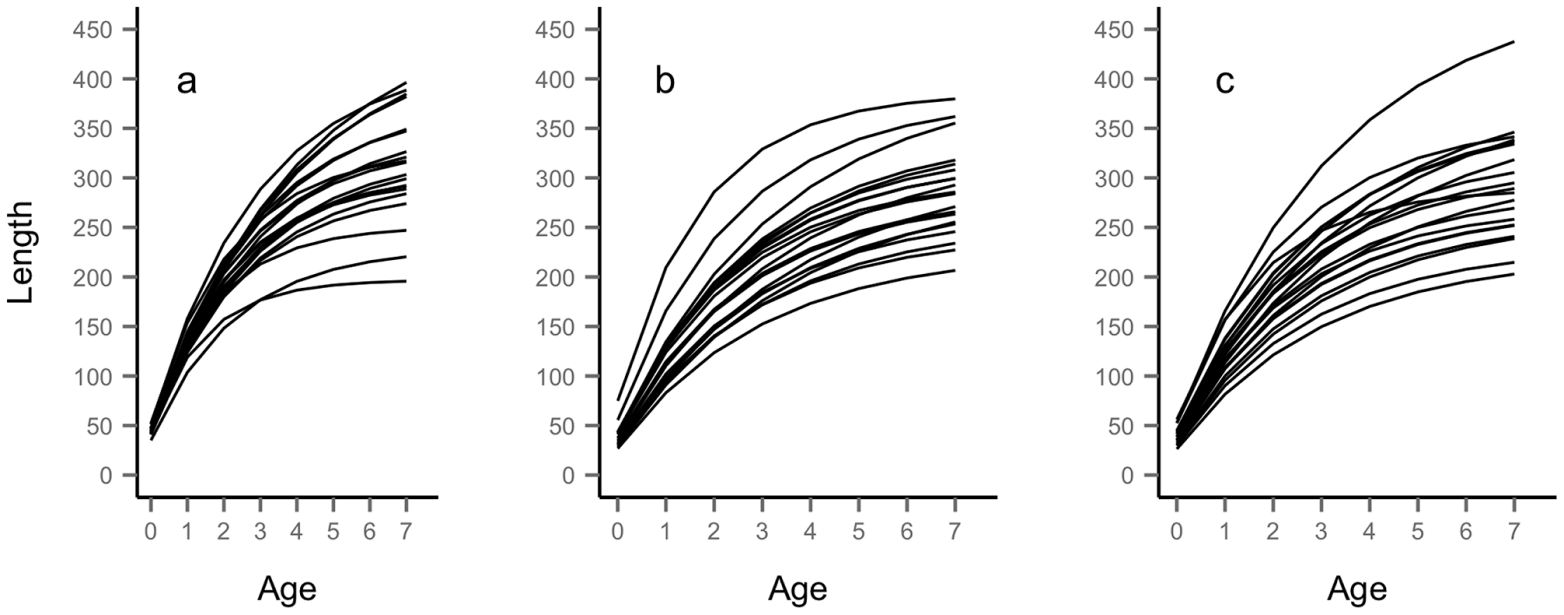
Growth trajectories from simulated data with negative, positive and no correlation between *L*_∞_ and *k*. Growth trajectories from simulated data according to Eq. 7 (no predictors, only intercept and individual random effects) with strong negative (left panel, Pearson's *r* = −0.9), positive (middle panel, *r* = 0.6), and no correlation (right panel, *r* = 0) between *L*_∞_ and *k* at the individual level. For all three panels, *L*_∞_ = 330 mm, *k* = 0.37 y^−1^, *t*_0_ = −0.38 y, σ_v_ = 0.22, σ_u_ = 0.22.

According to Eq. 7, a positive correlation between 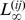 and 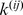 (from now on we will refer to them as *L*_∞_ and *k* at the individual level) indicates that size ranks tend to be maintained throughout the lifetime of individuals, while a negative correlation indicates that size ranks tend not to be maintained (fig. 2).

### Statistical analysis

#### Selection of best growth model

To begin, we were interested in the ability of the model with no predictors to describe the data used to calibrate the model (i.e. hindcasting). We checked the maximum gradient component to ensure that a satisfactory optimum was reached, and used *R* ^2^ and mean absolute error (MAE) as measures of goodness of fit. Unless otherwise noted, each model we tested included individual random effects and intercept for *L*_∞_ and *k*.

We introduced predictors as fixed effects to test whether they improved model performance. In particular, we included as predictors (*i*) population density in the first year of life of the fish (ind ha^−1^) as a continuous variable (*x_ij_* Eq. 7) [33], and (*ii*) cohort as a group (i.e. categorical) variable (α_1_ and *β*_1_ in Eq. 7). All fish in a cohort experience the same population density in the first year of life, thus we can intuitively think of the cohort effect as including other factors beyond early density affecting growth that are largely shared by the cohort, such as temperature at emergence/first stages of life (although the cohort effect is categorical, while the density effect is continuous).

We treated predictors as fixed effects for two reasons. First, introducing predictors as random effect is computationally more demanding in ADMB than using fixed effects, in the sense that run times for parameter estimation are substantially longer. This drawback is greatest when thousands of individuals are included in the dataset, as in the case of the marble trout population of Gacnik. Second, treating a factor with just a few levels as random factors may generate imprecise estimates of the associated standard deviation [52].

For each population, we fitted models in which density- or cohort effects were introduced in *k* or *L*_∞_. For simplicity and ease of interpretation, in each model we introduced at most one predictor for the two vBGF parameters (table 1). To guard against inconsistent parameter estimates caused by likelihood functions with multiple maxima, we started ADMB-RE from different initial parameter values and checked for consistency of parameter estimates.

**Table 1:** AIC of tested models.

	Models								
	No pred	*L*_∞_ = f(*D*)	*L*_∞_ = f(*coh*)	*k* = f(*D*)	*k* = f(*coh*)	*k* = f(*coh)*	*k* = f(*coh*)	*k* = f(*D*)	*k* = f(*D*)
						*L*_∞_ = f(*coh*)	*L*_∞_ = f(*D*)	*L*_∞_ = f(*D*)	*L*_∞_ = f(*coh*)
Zakojska	18171.6	18165.1	18116.7	18155.1	18114.9	18076.9	18113.2	18152.46	18103.8
	(6, 94.7)	(7, 88.2)	(16, 39.8)	(7, 78.2)	(16, 38.0)	(26, 0)	(17, 36.3)	(8, 75.6)	(17, 26.9)
Gacnik	82742.4	82207.8	81954.8	82152.0	81906.0	81598.0	92114.0	82151.6	81890.6
	(6, 1144.4)	(7, 609.8)	(16, 356.8)	(7, 554)	(16, 308)	(26, 0)	(17, 10516)	(8, 553.6)	(17, 292.6)

AIC of tested models (*coh* = cohort, *D* = density of fish in the first year of life). For both marble trout populations of Zakojska and Gacnik, the best model had cohort as predictor for both 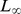 and *k* in addition to individual random effects. No pred = model with no predictors for either parameter. In parentheses are reported the number of parameters of the model and the ΔAIC with respect to the best model.

We used the Akaike Information Criterion [74], [75] to select the best model, although we also tested consistency of AIC ranking against the Bayesian Information Criterion (BIC) [76]. Following [58], we also tested whether a log transformation of population density decreased AIC of models that had density as predictor, but results were basically unaffected by the log transformation.

We then investigated correlation between the EB estimates of 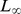 and *k* at the individual level and of cohort-specific mean 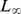 and *k* when cohort was a predictor of both 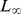 and *k* (e.g. following Eq. 7, 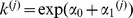). We used simulated data to test whether a significant correlation may emerge as an artifact of the algorithm for parameter estimation. Specifically, we simulated growth data with a randomly drawn correlation *r* between 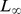 and *k* at the individual or cohort level and we then tested whether the empirical correlation between estimates of 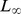 and *k* at the individual or cohort level obtained using the model fitting procedure in ADMB-RE was equal (or very close) to *r*.

We also tested whether there were noticeable differences in vBGF cohort-specific models when estimating parameters separately for each cohort using a standard non-linear regression routine with no random effects (*nls* function in R [77]) or using ADMB-RE. We carried out this analysis in order to determine whether the fitting of a random-effects model is recommended even when only mean growth trajectories at the group level are needed, thus in the case when the fitting of a standard non-linear regression model may represent a theoretically viable procedure.

#### Predicting missing data

We tested the predictive ability of the best vBGF model (after AIC selection) as follows. For each population, we: (*i*) identified fish that were sampled more than 3 times; (*ii*) randomly sampled one third of them (validation sample); (*iii*) deleted from the data set all observations except the first one from each individual fish in the validation sample; (*iv*) estimated the parameters of the vBGF for each individual including those in the validation sample; and (*v*) predicted the missing observations.

We compared the predictions of the vBGF to the predictions given by the mean length-at-age of fish in the population, including information given by predictors if included in the best model (e.g. if cohort was included as predictor in the best model, mean length-at-age of the fish cohort was used for prediction). We used MAE and *R* ^2^ of the 1∶1 predicted-observed line as measures of predictive ability. We tested the predictive abilities of the best vBGF model using 20 random validation samples for each population. In addition, we tested the predictive abilities of the vBGF model without predictors.

### Supplementary information and code

In Supporting Information we provide (*i*) tests of correlation between individual and mean cohort-specific 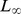 and *k* using simulated datasets (fig. S1 and table S3), (*ii*) the mean estimate and confidence intervals for parameters of the best models (table S2 and table S3), (*iii*) cohort-specific growth trajectories (fig. S2), (*iv*) derivation of the correlation between parameters of the vBGF under size-dependent mortality and description of potential processes leading to a negative correlation between 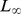 and *k* (text S1), (*v*) confidence bands estimated using a MonteCarlo algorithm (fig. S3), (*vi*) a comparison with JAGS and the *nlme* function in R (text S2), (*vii*) results of a repeatability analysis of body size throughout the lifetime [78], [79] (text S3), and (*viii*) details of the Empirical Bayes algorithm (text S4). All data and code used for the analyses and to produce figures can be found in an online repository at http://dx.doi.org/10.6084/m9.figshare.831432.

## Results

The empirical growth trajectories showed substantial individual variation in growth of marble trout in both populations (fig. 1). For each age-class except age-1, the Zakojska marble trout population had greater mean length than the Gacnik population (*p*<0.01 for all age-specific *t*-tests, results provided in the online repository). The mean length of age-1 fish was significantly greater in Gacnik (Welch's *t*-test: *t* = 5.28, *df* = 908.02, 95% CI = 2.13–4.66 mm, *p*<0.01). Maximum length reached by a fish was 396 mm at age 8 in Zakojska and 457 mm at age 12 in Gacnik.

### Estimates of parameters

For each vBGF model we tested, we obtained convergence of the algorithm for parameter estimation in ADMB, and the data used for the estimation of the parameters were well predicted by the models (for the model with no predictors except individual random effects: Zakojska, *R* ^2^ = 0.97, MAE = 9.58 mm; Gacnik, *R* ^2^ = 0.98, MAE = 6.82 mm).

**Figure 3:**
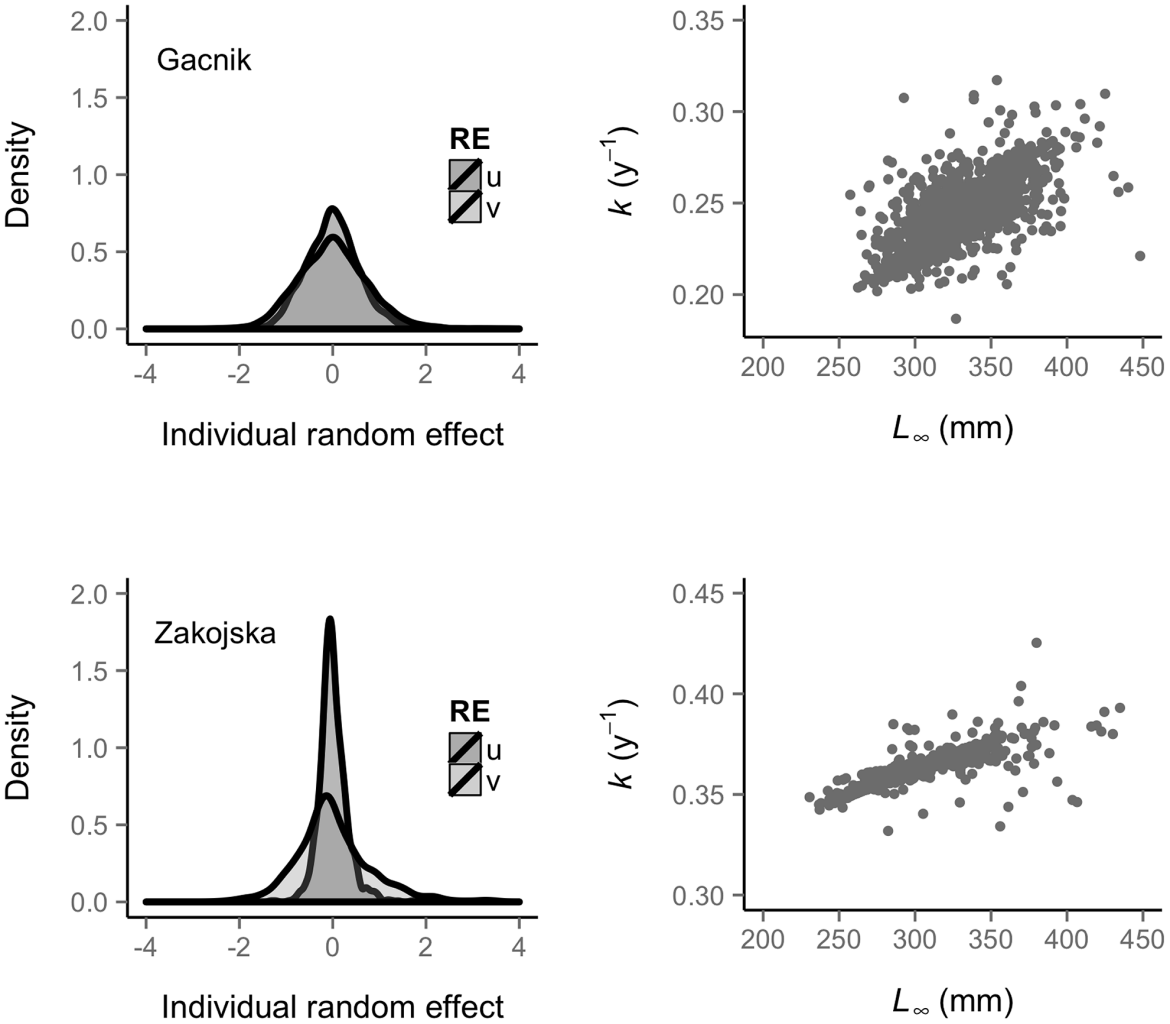
Distribution of estimates of individual random effects and correlation between *L*_∞_ and *k*. Distribution of estimates of individual random effects (left column, *u* = random effect for *k*, *v* = random effect for 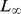) and plot of individual-level 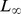 (mm) and *k* (y^−1^) (right column) for the populations of Zakojska (top row) and Gacnik (bottom row). For Zakojska: σ*_u_* = 0.06, σ*_v_* = 0.11; for Gacnik, σ*_u_* = 0.10, σ*_v_* = 0.09.

We obtained consistent parameter estimates when starting ADMB-RE from different initial parameter values. For each model, the standard deviation of the probability distribution of random effects was larger than 0. In the vBGF model with no predictors for both 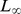 and *k*, the two parameters at the individual level were strongly and positively correlated (Zakojska; *r* = 0.79, *p*<0.01; Gacnik, *r* = 0.85, *p*<0.01) (fig. 3). However, the correlation was inflated by the almost perfect correlation of *k* and 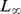 for fish that were sampled just once (Zakojska; *r* = 0.97, *p*<0.01; Gacnik, *r* = 0.99, *p*<0.01). Considering only fish that were sampled more than 2 times, the correlation between *k* or 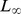 at the individual level remained positive and highly significant in both populations, albeit weaker (Zakojska; *r* = 0.48, *p*<0.01; Gacnik, *r* = 0.59, *p*<0.01). We also found a strong and positive correlation within cohorts between *k* and 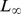 at the individual level in the models that included cohort as predictor in either or both parameters (for the model with cohort as predictor for both *k* and 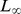, Zakojska [mean *r* across cohorts ± sd] = 0.86±0.11; Gacnik = 0.86±0.12). Tests on simulated data sets showed that when individual trajectories are simulated with positive, negative or no correlation *r* between *k* and 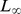 at the individual level, the estimated correlation between individual random effects estimated with the EB method is very close to the true *r* (fig. S1). The CVs of *k* and 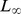 at the individual level for the vBGF model with no predictors were 6% and 6% respectively in Gacnik and 2% and 9% respectively in Zakojska. When the model included cohort as predictor for both *k* and 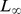, the range of cohort-specific CV of *k* and 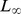 at the individual level were 3–6% (*k*) and 4–7% (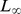) for Gacnik and 1–2% (*k*) and 3–13% (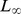) for Zakojska. In the model with no predictors, 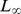 at the population level was greater in Gacnik than in Zakojska, while the opposite was true for *k* (mean and 95% confidence intervals, Gacnik: *L*_∞_ = 323.28 mm [318.54–328.02], *k* = 0.24 y^−1^ [0.23–0.25], *t*_0_ = −0.92 y [−0. 97-(−0.87)]; Zakojska: *L*_∞_ = 298.83 mm [289.83–307.82], *k* = 0.36 y^−1^ [0.33–0.39], *t*_0_ = −0.49 y [−0.58-(−0.41)]).

**Figure 4:**
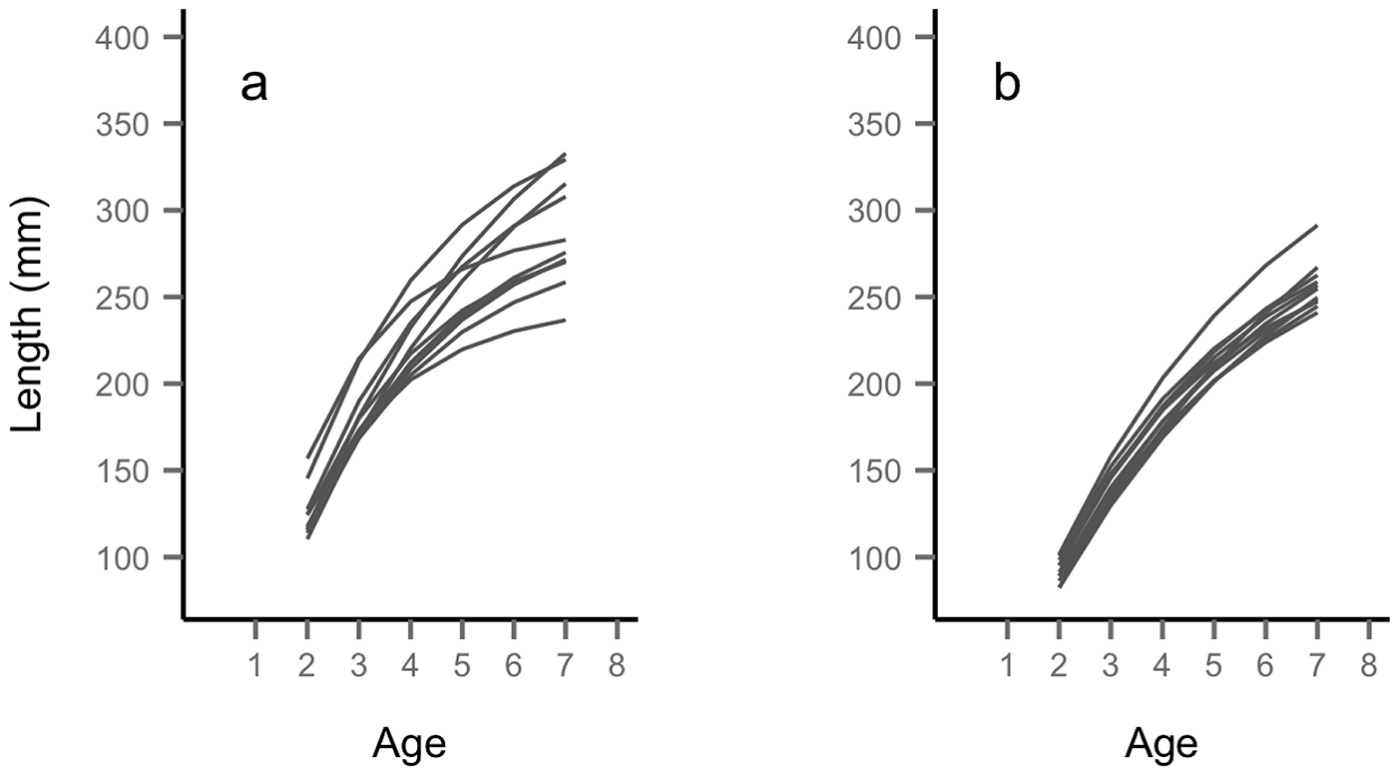
Cohort-specific growth trajectories. Cohort-specific growth trajectories for the marble trout populations of Zakojska (panel a) and Gacnik (b). The cohorts with the biggest and smallest body size at age 8 as predicted by the model were for Zakojska the 2006 and 2004 cohorts and for Gacnik the 2000 and 2003 cohorts.

For both populations, *k* and 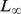 tended to get smaller with increasing density in the first year of life. The best model according to AIC had cohort as predictor of both *k* and 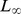 in both populations (table 1, see table S1 and S2 for parameter estimates for Zakojska and Gacnik, respectively). Cohort-specific mean *k* and 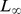 (i.e., with individual random effects *u*_ij_ and *v*_ij_ in Eq. 7 set to 0) were negatively correlated (Zakojska; *r* = −0.81, *p*<0.01; Gacnik, *r* = −0.87, *p*<0.01) (figs. 4 and S2). Simulations showed that the estimated correlation between mean cohort-specific *k* and 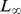 is not an artifact of the parameter estimation procedure (table S3).

**Figure 5:**
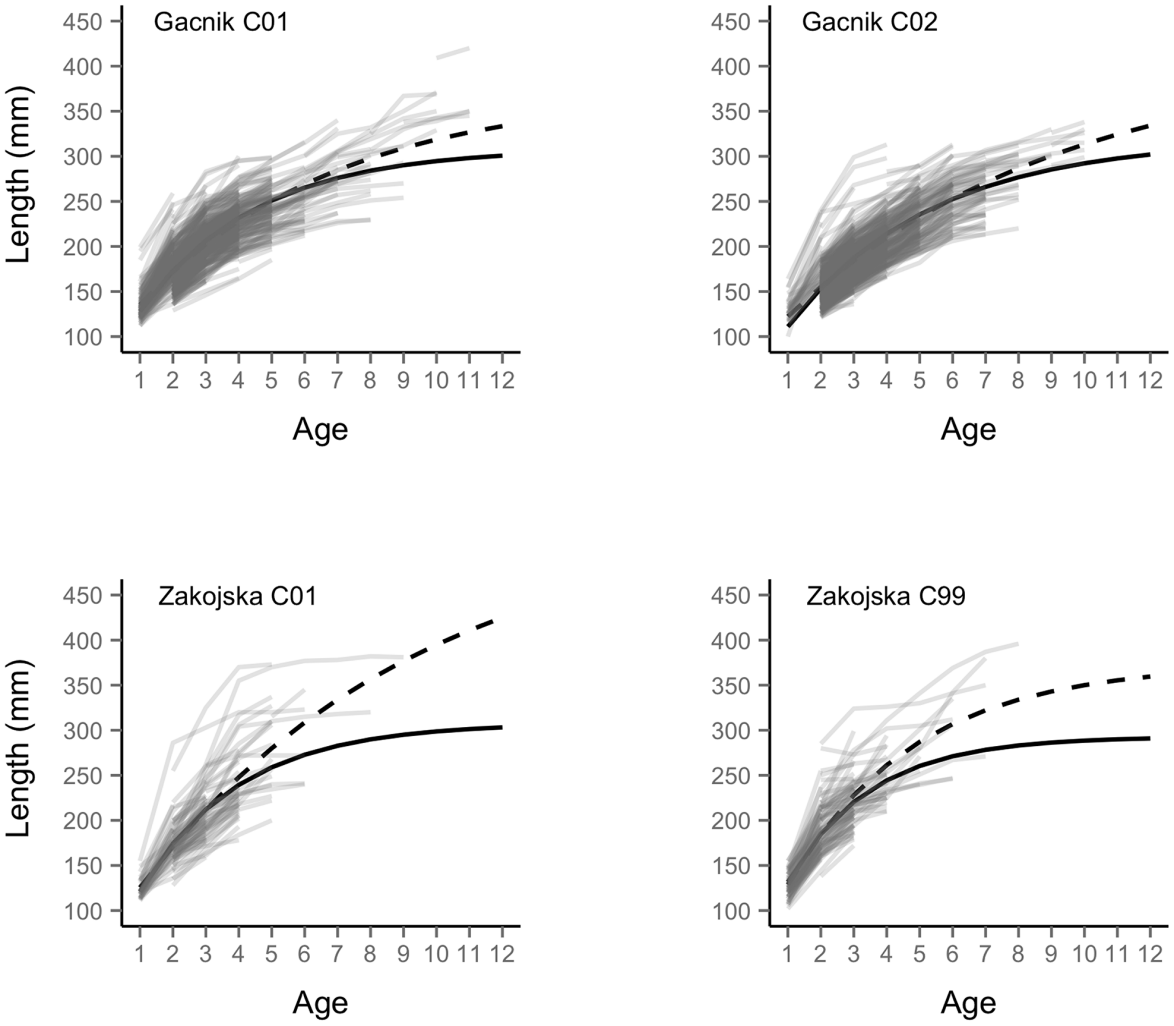
Prediction of mean cohort-specific growth with random-effects or non-linear least squares model. Prediction of mean cohort-specific growth trajectories (i.e. individual random effects *u* and *v* = 0) using the von Bertalanffy growth function model with cohort as a categorical predictor for both 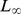 and *k* (solid line) and non-linear least-squares regression using the R function *nls* (dashed line) for the 2001 (a) and 2002 (b) cohorts for the population of Gacnik, and 2001 (c) and 1999 (d) cohorts for the population of Zakojska. Estimates of model parameters are reported in Table 3.

Cohort-specific models with no random effects (i.e. parameters estimated using *nls* function in R) provided consistently greater estimates of 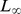 and smaller estimates of *k* than random-effects models (fig. 5 and table 2), which showed that ignoring autocorrelation among individual measures is likely to upwardly bias estimates of asymptotic length at the group level.

**Table 2:** Parameters of the von Bertalanffy growth function model for two cohorts of Gacnik and Zakojska.

	*nls*			*random-effects*		
Cohort	*L*_∞_ (mm)	*k* (y^−1^)	*t_0_* (y)	*L*_∞_ (mm)	*k* (y^−1^)	*t_0_* (y)
Gacnik 2001	370.34[350.91–394.93]	0.17[0.14–0.19]	−1.70[−1.95-(−1.47)]	308.51[303.06–313.96]	0.29[0.27–0.30]	−0.87[−0.92-(−0.83)]
Gacnik 2002	410.15[376.03–459.84]	0.12[0.10–0.15]	−1.93[−2.28-(−1.62)]	318.87[312.38–325.36]	0.23[0.22–0.24]	−0.87[−0.92-(−0.83)]
Zakojska 2001	539.61[424.46–870.87]	0.11[0.05–0.18]	−1.27[−1.85-(−0.83)]	307.88 [293.64–322.12]	0.33[0.30–0.37]	−0.48[−0.57-(−0.41)]
Zakojska 1999	373.55[335.65–434.51]	0.26[0.19–0.34]	−0.62[−0.95-(−0.36)]	291.98[262.36–321.59]	1.27[1.18–1.36]	−0.48[−0.57-(−0.41)]

Parameter estimates and 95% confidence intervals of the von Bertalanffy growth function model for two cohorts of Gacnik and Zakojska with individuals random effects and cohort as predictors for both 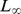 and *k* (random-effects model) and non-linear least squares regression separately for each cohort using the R function *nls*. 95% confidence intervals of parameters estimates for the two models do not overlap for any of the von Bertalanffy growth function's parameters.

### Prediction of lifetime growth trajectories

**Figure 6:**
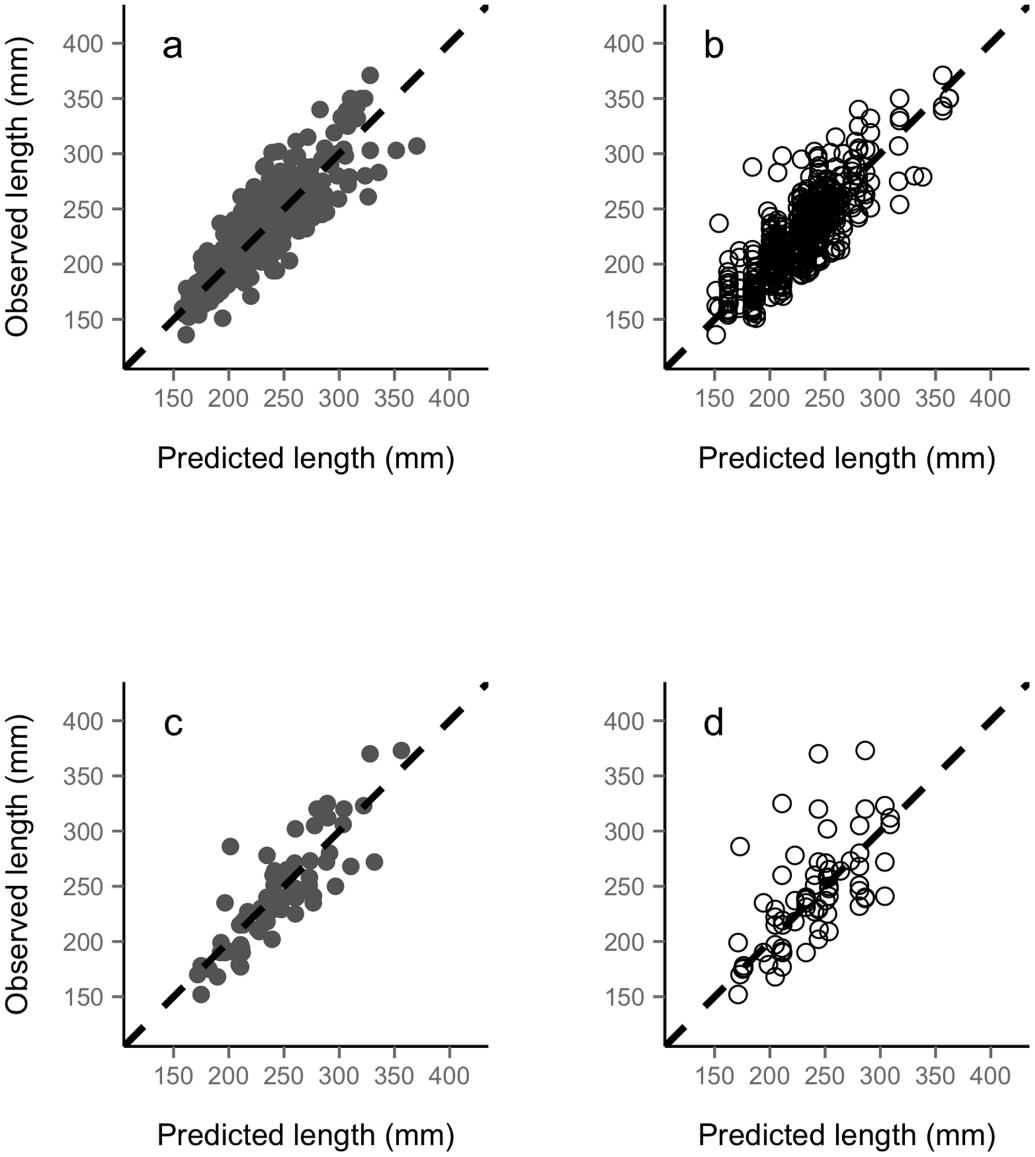
Prediction of validation data using random-effects model or mean cohort-specific length-at-age empirical data. Example of prediction of validation data for the population of Gacnik using the model with cohort as predictor for both 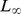 and *k* (panel a, *R* ^2^ = 0.76, MAE = 19 mm) and mean cohort-specific length-at-age empirical data (b, *R* ^2^ = 0.66, MAE = 22 mm). For the population of Zakojska, (c) model predictions (*R* ^2^ = 0.72, MAE = 24 mm), and (d) predictions using mean cohort-specific length-at-age empirical data (*R* ^2^ = 0.36, MAE = 37 mm).

In the populations of Gacnik and Zakojska 450 and 62 fish respectively have been sampled more than 3 times during their lifetime. For both populations, the best vBGF model (i.e. model including cohort and individual random effects as predictors for both *k* and 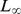) fitted for the fish in the validation samples using only the first observation (20% of 450 and 62 fish for Gacnik and Zakojska, respectively) provided better prediction of the missing observations than mean length-at-age of the respective fish cohort (fig. 6, table 3). Finally, when we used no predictors for either model parameter except the individual random effects, the random-effects model provided better predictions of the missing observations than population mean length-at-age (table 3).

**Table 3:** Prediction of future growth trajectories.

	Best model				Model with no predictors			
Population	Model		Cohort mean length		Model		Population mean length	
	*R* ^2^	MAE	*R* ^2^	MAE	*R* ^2^	MAE	*R* ^2^	MAE
Zakojska	0.63±0.08	29.87±6.07	0.51±0.10	34.27±4.25	0.58±0.09	31.63±6.15	0.36±0.14	38.80±4.25
Gacnik	0.80±0.02	17.62±0.84	0.64±0.04	23.63±1.03	0.77v0.02	18.78±0.85	0.50±0.04	27.54±1.43

Mean ± sd of *R* ^2^ and mean absolute error (MAE, mm) of predictions of validation data as provided by the vBGF model including cohort as predictor for both *k* and 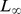 at the individual level (best model) and by the vBGF model with no predictors for either parameter for 20 random validation samples. In both cases, we also report mean ± sd of *R* ^2^ and MAE of predictions with mean length-at-age of the respective cohort (for the model with cohort as predictor for both in *k* and 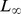) and of the population as a whole (for the model with no predictors) for the same validation samples.

## Discussion

The Empirical Bayes approach applied to the estimation of a parameter-rich non-linear growth function including individual random effects provides a computationally efficient methodology to estimate shared and individual variation in growth. Other methods and routines can be applied to the estimation of random-effects non-linear models of growth, for instance the *nlme* function in R or BUGS/JAGS. However, as we report in text S2 and in the online code, when dealing with a large number of random effects, missing data, or “noisy” growth of individuals, some of those methods may take a very long time to converge or fail to converge. By providing a general template for fitting growth curves (i.e. not limited to the von Bertalanffy growth function) with ADMB-RE, our goal is to encourage and help researchers using more sophisticated tools to obtain fast and reliable parameter estimates of non-linear random-effects growth models using longitudinal or back-calculated data.

We now discuss our results on the determinants of growth of marble trout, as well as how the results obtained through the application of the Empirical Bayes approach lead to hypotheses on life-history strategies and on the interplay between genetic and environmental determinants of some of marble trout life histories.

### Maintenance of size ranks and correlation between *L*_∞_ and *k*

As described above, we found a strong positive correlation between 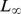 and *k* at the individual level, as well as very high repeatability of body size in both populations (text S3). These two results concordantly indicate that size ranks are strongly maintained over time.

Two other studies investigated the correlation between the von Bertalanffy growth function's parameters 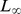 and *k* at individual level. Using a random-effects model implemented in BUGS, Pilling et al. [80] found a strong negative correlation between 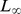 and *k* at the individual level in a sky emperor *Lethrinus mahsena* population, but they did not discuss any potential processes leading to the estimated negative correlation. In [81], Alós et al. using a modified five-parameter von Bertalanffy growth function implemented in BUGS found a positive correlation between 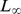 and two growth parameters (*k*_0_ and *k*_1_) at the individual level, but they did not discuss the biological and ecological determinants of the observed positive correlation among parameters of the growth function. In text S1, we discuss the processes that may lead to a negative correlation between 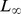 and *k* and here focus on the positive correlation.

At the population or group level, the correlation between 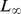 and *k* obtained from the Hessian estimated at maximum likelihood estimates of the parameters is usually negative. This correlation does not offer any biological insights, since it occurs because different combinations of 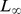 and *k* can basically provide the same fit to the data, in particular when the range of ages is limited [58], [82], [83]. In other words, by slightly increasing or decreasing 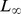 and *k* in opposite directions, the same likelihood is obtained. Although it is possible to estimate the correlation between random effects within ADMB-RE, this may lead to computational instabilities and possibly to ambiguous interpretation of the correlation parameter when other predictors are taken into account (we provide the code in the online repository). Our simulations confirmed that the observed positive correlation between estimates of 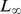 and *k* at the group level (cohort, as in our case) and at the individual levels is not a statistical artifact.

Multiple non-exclusive and potentially interacting processes may lead to the maintenance of size ranks throughout marble trout lifetime. Specifically, we consider three potential processes: (*i*) among-fish differences in genetic growth potential; (*ii*) habitat heterogeneity; (*iii*) size-dependent piscivory.

#### Differences in genetic growth potential

Some fish may have consistently greater growth performance either due to more efficient resource acquisition, different endocrine regulation (e.g. growth hormone – insulin-like growth factor I axis) [84] or preferential allocation of energy to growth. Heritability (*h* ^2^) is the proportion of phenotypic variance explained by additive genetic variance [85]. Although estimates of *h* ^2^ for size-at-age for marble trout are currently not available, the success of artificial selection for improved growth traits in fish farms for salmonids (13% increase in body size-at-age per generation in Atlantic salmon [86], [87]) as well as available empirical estimates of heritability of length-at-age in the wild (median *h* ^2^ = 0.29) [23] confirm the existence of substantial additive genetic variance for growth in salmonids.

In [79], Letcher et al. found, for a population of brook trout living in West Brook (MA, US), that most of the observed size variation among fish derived from size differences at the juvenile stage (age-0 fish), which they assumed were determined by heritable differences in the timing of emergence. Since size variation throughout the lifetime of brook trout was only moderately influenced by subsequent size-dependent processes, size ranks were largely maintained over time. Letcher et al. [79] argued that the most likely mechanism for maintaining size ranks in salmonids is the establishment of dominance hierarchies [88]–[90], which may translate to the occupation of sites of different profitability.

Repeatability sets an upper limit to heritability (but see [91]), and a large difference between repeatability and heritability for a trait may suggest that the trait is at least partially determined by environmental (including trophic) conditions causing variation mostly independent from genotypes. The very high estimates of repeatability for length-at-age we found for both populations (Gacnik: mean and 95% credible intervals = 0.75 [0.73–0.76]; Zakojska = 0.66 [0.62–0.70], see text S3) and the median estimate of heritability for length-at-age in salmonids suggest that a large amount of variation in individual growth trajectories is determined by environmental factors, and this is likely to limit the evolution of growth rates following episodes of massive mortality of marble trout [22], [24].

#### Habitat heterogeneity

A patchy distribution of resources can potentially lead to the maintenance of size ranks throughout fish lifetime. That is, due to abiotic (e.g. water velocity, turbidity, size and location of shelter, micro-variation in temperature) and biotic (e.g. availability and type of prey) factors, some portions of the stream habitat are more profitable than others [34]. While it is obvious that a greater abundance and energetic content of prey – as well as optimal temperature [92] - increase the potential for growth, slow currents decrease the energy expenditure for maintaining position [93]. On the other hand, in the case of fish that are mobile and can potentially explore or occupy different parts of the stream, the positive correlation between 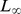 and *k* due to heterogeneous site profitability may or may not emerge.

However, marble trout living in Gacnik and Zakojska rarely move more than two hundred meters throughout their lifetime [94] and the existence of areas of different profitability can be easily inferred by the consistent bigger size-at-age of fish occupying the uppermost part of the stream (i.e. where a larger portion of stream drift is available since no fish are present upstream) than of fish living further downstream [94].

#### Size-dependent piscivory

In the presence of growth variation among fish early in life, size-dependent piscivory [95] may generate a positive feedback process on growth and body size-at-age, in which fish growing faster early in life both (*i*) reach the size threshold for piscivory and (*ii*) are able to eat larger prey earlier than fish growing more slowly [96]–[98]. Marble trout are cannibalistic in mountain streams, and preliminary isotopic analyses indicate that the initiation of cannibalism is size dependent and usually starts at age 3 years old.

### Best models for the marble trout populations of Zakojska and Gacnik

The best model for both populations included cohort as a categorical predictor for both 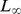 and *k*. Within each cohort we found substantial individual variation as well as strong maintenance of size ranks throughout marble trout lifetime (i.e. the within-cohort correlation of 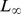 and *k* at the individual level was strongly positive). Models including only density in the first year of life performed distinctly worse than the best model, but better than the model with no predictors. This seems to suggest that other factors, in addition to early density experienced by cohorts, contribute to determine mean growth trajectories of cohorts. Apart from climatic vagaries or particular trophic conditions affecting cohorts in their early life stages, another possible explanation for the emergence of cohort effect is high variance in reproductive success (e.g. just a few fish contribute to the next generation), which is common in salmonids [99], [100], combined with (*i*) high heritability of growth and/or (*ii*) heterogeneity in site profitability accompanied by limited movement. The mean growth trajectory of the cohort may thus signal in case of (*i*) the growth potential of the small parental pool, or in case of (*ii*) the profitability of the stream habitat where a large fraction of the cohort lived.

Cohort effects on growth were more pronounced in Zakojska than in Gacnik. We found a strong negative correlation between cohort-specific mean 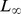 and *k* in both populations. Thus, some of the mean growth trajectories of cohorts were crossing throughout fish lifetime, but within cohorts size ranks were mostly maintained over time. However, the cohort-specific growth trajectories in Gacnik showed very little variation with the exception of a particularly fast-growing cohort, while a richer variety of cohort-specific mean growth trajectories were observed in Zakojska. This may be in part related to the estimation of 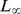 being particularly sensitive to the presence in the dataset of older individuals [101]. In Zakojska, the dramatic reduction in population size after the flood of 2007 accompanied by the natural thinning of cohorts over time reduced the number of older individuals in the dataset, and this may lead to less accurate predicted mean size of cohorts at older ages. However, we observed the same strong negative correlation even if only including cohorts born up to 2002 (fig. S2), although the diversity of cohort-specific growth pattern was noticeably smaller and comparable to the diversity observed in Gacnik.

Fast-growing cohorts can play a key role in the persistence of small fish populations. Since sexual maturity and egg production in fish are generally size dependent [102], a higher proportion of fish can reach sexual maturity at younger ages in a fast-growing cohorts than in slow-growing ones. This may be crucial when population size is low and the population is at risk of extinction due to demographic stochasticity. In both Gacnik and Zakojska, the fastest-growing cohorts experienced very low population densities in the first two years of life. Further studies should test whether at the individual or at the cohort level a trade-off between growth and mortality can be observed [103], and whether fast-growing cohorts had higher lifetime reproductive success than slow-growing ones.

### Prediction of growth trajectories

A sizable literature on prediction of future growth exists for humans, especially in the context of early identification of pathologies [104]–[107]. An approach similar to that presented in our work for the estimation of lifetime growth trajectories given only information on growth and size during the early stages of life was proposed in [104] and [105]. In particular, in [104] Shohoji et al estimated the lifetime growth of Japanese girls using measurement up to the age of 6 years old. They first adapted to humans a parametric model previously developed to model the growth in weight of savannah baboons. Then, they tested the suitability of an Empirical Bayes approach to estimate model parameters and predict abnormal growth at later stages of life. They found that classification of individuals into proper homogenous groups (i.e. where the strength is borrowed from) was necessary in order to obtain accurate predictions of lifetime growth. In [105], Berkey found that there is a point beyond which the Empirical Bayes method (but more in general any method) is no longer robust to missing data, and found - as expected - that growth curve parameters are especially sensitive to the end points of the growth trajectories.

Given an appropriate growth model, the prediction of lifetime growth trajectories from early measurements presents further complications - as in our case - when dealing with organisms that still grow after sexual maturity [108] and when homogenous groups (i.e. cohorts) may include just a few individuals reaching older ages. In addition, when using the vBGF model with both 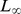 and *k* function of cohort and individual random effects, the estimation of cohort effects should be robust to the deletion from the dataset of one-third of the individuals that have been sampled more than 3 times, since the presence of only a few old individuals in the dataset is likely to bias the estimation of 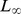 [101].

Our results indicate that when strength is borrowed from other individuals, parameters estimated on a single measurement can be used to summarize the growth trajectory of marble trout living in Zakojska and Gacnik and to impute missing observation for the estimation of size-dependent survival. The best vBGF model provided predictions of future growth trajectories in both populations that were consistently (i.e. for all validation samples) better than simply using the mean length-at-age of the fish cohort. Clearly, other covariates presently not available or not included in the model, such as sex or position in the stream, may help further improve predictions of lifetime growth and size-at-age.

We found better predictions across validation samples for the population of Gacnik than for the Zakojska population. This may be due to a higher number of fish both overall and in each cohort in Gacnik, less variability in growth at the whole population level as well as among fish in the same cohort, as evidenced by the much smaller coefficient of variation of 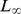 and higher repeatability of body size in Gacnik than in Zakojska, or a lower plasticity of growth trajectories after the first year of life in Gacnik than in Zakojska, which may be caused by more homogenous site profitability in Gacnik.

In conclusion, in this work we have shown how the estimation of parameters of a parameter-rich non-linear growth function using longitudinal data can shed light on the shared and individual determinants of somatic growth in natural populations. The estimation method based on the Empirical Bayes approach is readily applicable to different parameterizations of the von Bertalanffy growth function or other growth models, and it provides additional flexibility, speed and ease of use with respect to other approaches [8]. In the case of more frequent sampling of individuals [10], models with seasonal components may be used and the inclusion of more fine-grained candidate predictors (such as monthly temperature, flow, trophic conditions) when available may be tested.

## Supporting Information

Figure S1 **Correlation between random effects.** Correlation between random effects *u* and *v* in the random-effect vBGF model (see Eq. 7 in the main text). Points are the simulated data, *r*_R_ is the Pearson's correlation on the simulated data, *r*_E_ is the estimated correlation of *u* and *v* estimated by the Empirical Bayes method.
(PDF)

Figure S2 **Cohort-specific growth trajectories.** Cohort-specific growth trajectories for the marble trout populations of Zakojska for cohorts up to 2002 included.
(PDF)

Figure S3 **Confidence bands for mean cohort-specific growth trajectory.** Confidence bands for mean cohort-specific growth trajectory (i.e. random effects *u* and *v* = 0) using the von Bertalanffy growth function model with cohort as a categorical predictor for both *L*_∞_ and *k* (solid line) and non-linear least-squares regression using the R function *nls* (dashed line) for the 1999 cohort of the Zakojska population (*nls* vBGF, mean and 95% confidence interval: *L*_∞_ = 373.55 mm [335.65–434.51], *k* = 0.26 y^−1^ [0.19–0.34], *t*_0_ = −0.62 y [−0.95-(−0.36)]; random-effect vBGF: *L*_∞_ = 291.98 mm [262.36–321.59], *k* = 1.27 y^−1^ [1.18–1.36], *t*_0_ = −0.48 y [−0.57-(−0.41)]).
(PDF)

Table S1 **Best model for Zakojska.** Parameters (mean and 95% confidence interval) of the best von Bertalanffy model according to AIC with *L*_∞_(mm) and *k* (y^−1^) function of cohort for the population of Zakojska. For all cohorts, *t*_0_ = −0.49 y [−0.57-(−0.41)], σ*_u_* = 0.05[0.02–0.09], σ*_v_* = 0.10[0.09–0.11].
(PDF)

Table S2 **Best model for Gacnik.** Parameters (mean and 95% confidence interval) of the best von Bertalanffy model according to AIC with *L*_∞_(mm) and *k* (y^−1^) function of cohort for the population of Gacnik. For all cohorts, *t*_0_ = −0.87[−0.92-(−0.82)], σ*_u_* = 0.09[0.08–0.10], σ*_v_* = 0.079[0.075–0082].
(PDF)

Table S3 **Correlation of cohort-specific and of individual** ***L*** **_∞_ and** ***k*** **.** Pearson's correlation between realized (i.e. not from the Hessian matrix) estimates of cohort-specific and of individual *L*_∞_(mm) and *k* (y^−1^). We carried out 30 reproducible replicates. Coh.Sim = correlation between simulated cohort-specific mean *L*_∞_ and *k* (individual random effects set to 0); Coh. Est = correlation between cohort-specific mean *L*_∞_ and *k* estimated by ADMB (individual random effects set to 0); Ind.Real = correlation between simulated *L*_∞_ and *k* at the individual level; Ind.Sim = correlation between *L*_∞_ and *k* at the individual level estimated by ADMB-RE.
(PDF)

Text S1 **Processes leading to negative correlation between *L*_∞_ and *k* and how size-dependent survival generates a negative correlation between random effects.**
(PDF)

Text S2 **Comparison with JAGS and *nlme* function in R.**
(PDF)

Text S3 **Repeatability analysis.**
(PDF)

Text S4 **The Empirical Bayes algorithm.**
(PDF)
