# Supplementary material for: Determining Individual Variation in Growth and Its Implication for Life-History and Population Processes Using the Empirical Bayes Method
Source: PLoS Comput Biol. 2014 Sep 11;10(9):e1003828. doi: 10.1371/journal.pcbi.1003828 (PMC4161297; doi:10.1371/journal.pcbi.1003828)
Supplement: Figure S1 — Correlation between random effects. Correlation between random effects u and v in the random-effect vBGF model (see Eq. 7 in the main text). Points are the simulated data, r R is the Pearson's correlation on the simulated data, r E is the estimated correlation of u and v estimated by the Empirical Bayes method. (PDF) [file pcbi.1003828.s001.pdf]

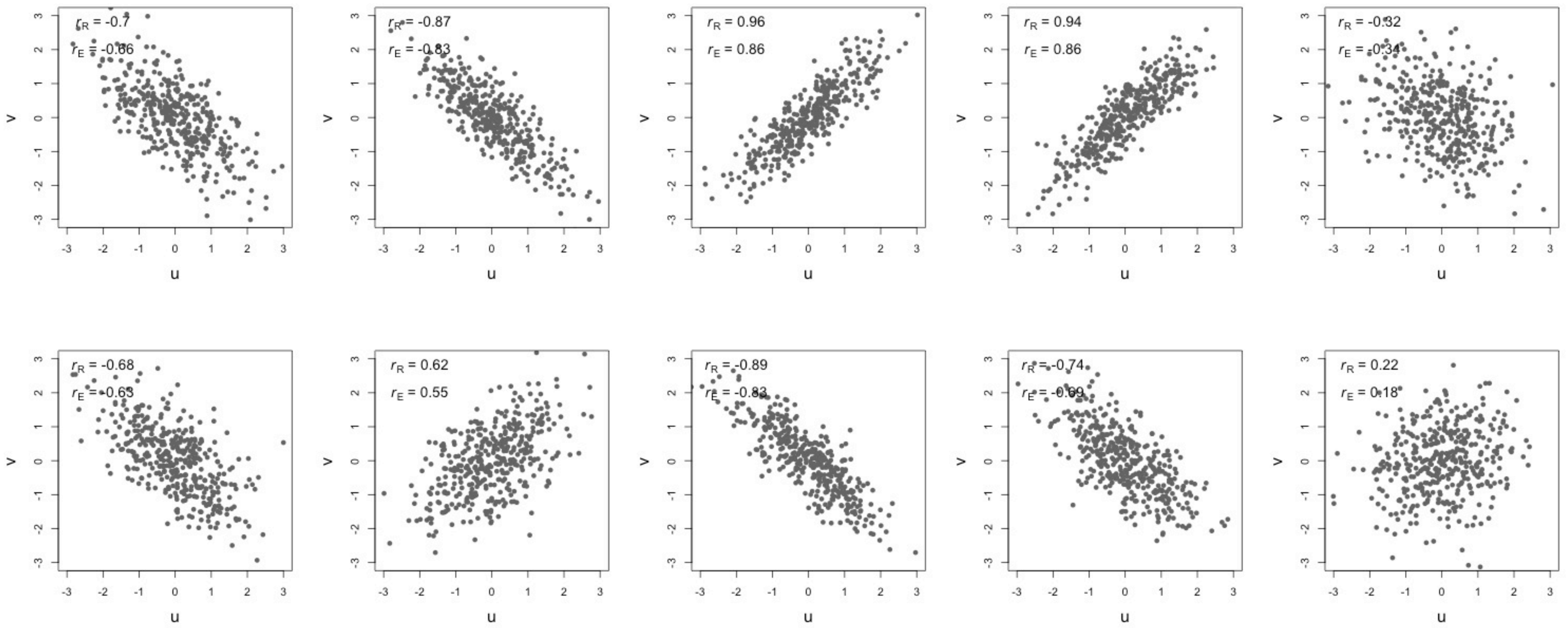


**Figure S1.** Correlation between random effects *u* and *v* in the random-effect vBGF model (see Eq. 7 in the main text). Points are the simulated data, $r_R$ is the Pearson's correlation on the simulated data, $r_E$ is the estimated correlation of *u* and *v* estimated by the Empirical Bayes method. Code for generating the figure is available at http://dx.doi.org/10.6084/m9.figshare.831432.
