# Supplementary material for: Determining Individual Variation in Growth and Its Implication for Life-History and Population Processes Using the Empirical Bayes Method
Source: PLoS Comput Biol. 2014 Sep 11;10(9):e1003828. doi: 10.1371/journal.pcbi.1003828 (PMC4161297; doi:10.1371/journal.pcbi.1003828)
Supplement: Figure S2 — Cohort-specific growth trajectories. Cohort-specific growth trajectories for the marble trout populations of Zakojska for cohorts up to 2002 included. (PDF) [file pcbi.1003828.s002.pdf]

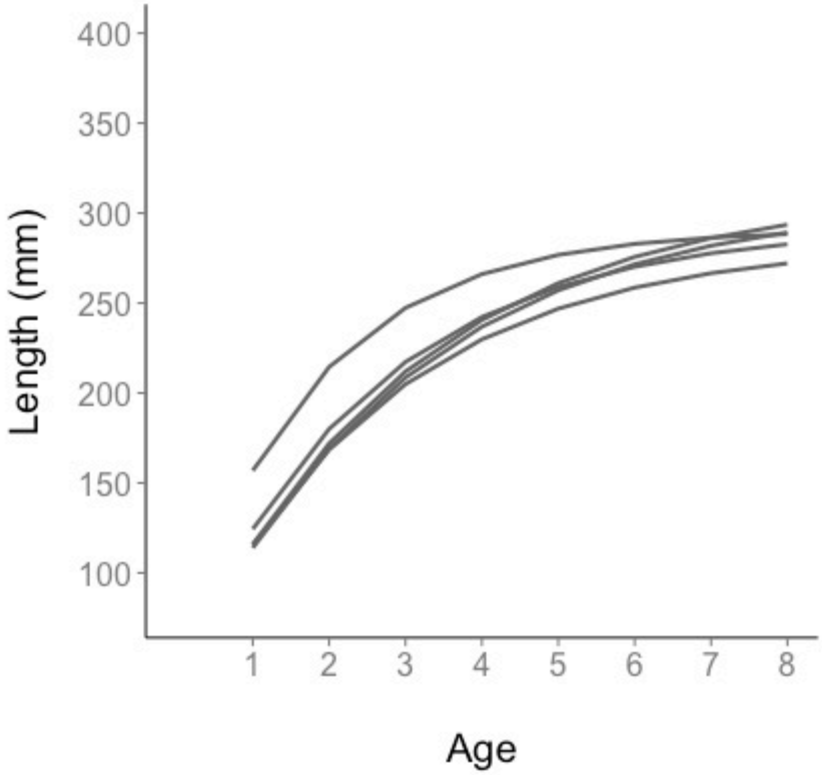

Figure S2. Cohort-specific growth trajectories for the marble trout populations of Zakojska for cohorts up to 2002 included.
