# Supplementary material for: Determining Individual Variation in Growth and Its Implication for Life-History and Population Processes Using the Empirical Bayes Method
Source: PLoS Comput Biol. 2014 Sep 11;10(9):e1003828. doi: 10.1371/journal.pcbi.1003828 (PMC4161297; doi:10.1371/journal.pcbi.1003828)
Supplement: Figure S3 — Confidence bands for mean cohort-specific growth trajectory. Confidence bands for mean cohort-specific growth trajectory (i.e. random effects u and v = 0) using the von Bertalanffy growth function model with cohort as a categorical predictor for both L ∞ and k (solid line) and non-linear least-squares regression using the R function nls (dashed line) for the 1999 cohort of the Zakojska population (nls vBGF, mean and 95% confidence interval: L ∞ = 373.55 mm [335.65–434.51], k = 0.26 y−1 [0.19–0.34], t 0 = −0.62 y [−0.95-(−0.36)]; random-effect vBGF: L ∞ = 291.98 mm [262.36–321.59], k = 1.27 y−1 [1.18–1.36], t 0 = −0.48 y [−0.57-(−0.41)]). (PDF) [file pcbi.1003828.s003.pdf]

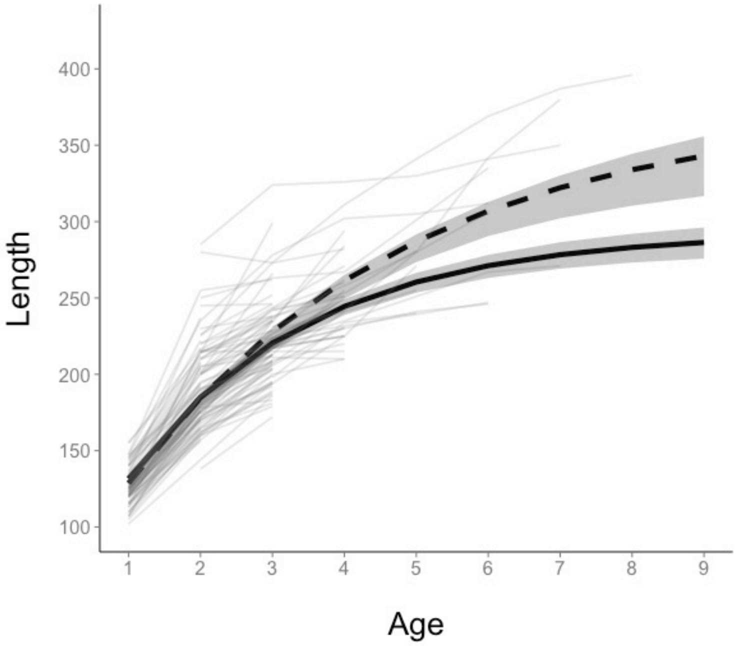

Figure S3. Confidence bands for mean cohort-specific growth trajectory (i.e. random effects *u* and *v* = 0) using the von Bertalanffy growth function model with cohort as a categorical predictor for both L_{∞} and *k* (solid line) and non-linear least-squares regression using the R function *nls* (dashed line) for the 1999 cohort of the Zakojska population (*nls* vBGF, mean and 95% confidence interval: L_{∞} = 373.55 mm [335.65-434.51], *k* = 0.26 y^{-1} [0.19-0.34], t_0 = -0.62 y [-0.95-(-0.36)]; random-effect vBGF: L_{∞} = 291.98 mm [262.36-321.59], *k* = 1.27 y^{-1} [1.18-1.36], t_0 = -0.48 y [-0.57-(-0.41)]).
