# Supplementary material for: Determining Individual Variation in Growth and Its Implication for Life-History and Population Processes Using the Empirical Bayes Method
Source: PLoS Comput Biol. 2014 Sep 11;10(9):e1003828. doi: 10.1371/journal.pcbi.1003828 (PMC4161297; doi:10.1371/journal.pcbi.1003828)
Supplement: Table S1 — Best model for Zakojska. Parameters (mean and 95% confidence interval) of the best von Bertalanffy model according to AIC with L ∞(mm) and k (y−1) function of cohort for the population of Zakojska. For all cohorts, t 0 = −0.49 y [−0.57-(−0.41)], σu = 0.05[0.02–0.09], σv = 0.10[0.09–0.11]. (PDF) [file pcbi.1003828.s004.pdf]

Table S1. Parameters (mean and 95% confidence interval) of the best von Bertalanffy model according to AIC with L_∞ (mm) and k (y^{-1}) function of cohort for the population of Zakojska. For all cohorts, t_0 = -0.49 y [-0.57-(-0.41)], σ_u = 0.05[0.02-0.09], σ_v = 0.10[0.09-0.11].

Cohort	L_∞ (mm)	k (y^{-1})
1998	290.98[261.26-320.70]	0.56[0.34-0.78]
1999	292.89[280.51-305.27]	0.40[0.36-0.44]
2000	284.00[271.65-296.34]	0.38 [0.34-0.41]
2001	307.88 [293.64-322.12]	0.33[0.30-0.37]
2002	312.23[287.92-336.54]	0.34[0.29-0.38]
2003	301.94[273.60-330.28]	0.36[0.30-0.42]
2004	246.20[225.06-267.34]	0.51[0.42-0.60]
2005	414.47[323.91-505.03]	0.22[0.16-0.29]
2006	437.44[224.07-650.81]	0.22[0.09-0.36]
2007	363.58[313.45-413.70]	0.37[0.28-0.46]
2008	351.76[288.83-414.68]	0.33[0.23-0.42]
