# Supplementary material for: Determining Individual Variation in Growth and Its Implication for Life-History and Population Processes Using the Empirical Bayes Method
Source: PLoS Comput Biol. 2014 Sep 11;10(9):e1003828. doi: 10.1371/journal.pcbi.1003828 (PMC4161297; doi:10.1371/journal.pcbi.1003828)
Supplement: Table S2 — Best model for Gacnik. Parameters (mean and 95% confidence interval) of the best von Bertalanffy model according to AIC with L ∞(mm) and k (y−1) function of cohort for the population of Gacnik. For all cohorts, t 0 = −0.87[−0.92-(−0.82)], σu = 0.09[0.08–0.10], σv = 0.079[0.075–0082]. (PDF) [file pcbi.1003828.s005.pdf]

Table S2. Parameters (mean and 95% confidence interval) of the best von Bertalanffy model according to AIC with L_{∞} (mm) and k (y^{-1}) function of cohort for the population of Gacnik. For all cohorts, t_0 = -0.87 y [-0.92-(-0.82)], σ_u = 0.09[0.08-0.10], σ_v = 0.079[0.075-0082].

Cohort	L_{∞} (mm)	k (y^{-1})
2000	384.55[371.03-398.07]	0.22[0.21-0.24]
2001	308.51[303.06-313.96]	0.29[0.27-0.30]
2002	318.87[312.38-325.36]	0.23[0.22-0.24]
2003	299.86[291.83-307.89]	0.25[0.24-0.27]
2004	334.85[321.33-348.37]	0.22[0.21-0.24]
2005	290.36[280.18-300.53]	0.30[0.28-0.32]
2006	318.76[304.36-333.16]	0.26[0.24-0.28]
2007	332.57[322.53-342.60]	0.24[0.23-0.26]
2008	420.87[391.60-450.14]	0.16[0.14-0.17]
2009	352.80[308.23-397.36]	0.19[0.16-0.23]
2010	309.31[217.26-401.35]	0.25[0.14-0.36]
