# Supplementary material for: Determining Individual Variation in Growth and Its Implication for Life-History and Population Processes Using the Empirical Bayes Method
Source: PLoS Comput Biol. 2014 Sep 11;10(9):e1003828. doi: 10.1371/journal.pcbi.1003828 (PMC4161297; doi:10.1371/journal.pcbi.1003828)
Supplement: Table S3 — Correlation of cohort-specific and of individual L ∞ and k . Pearson's correlation between realized (i.e. not from the Hessian matrix) estimates of cohort-specific and of individual L ∞(mm) and k (y−1). We carried out 30 reproducible replicates. Coh.Sim = correlation between simulated cohort-specific mean L ∞ and k (individual random effects set to 0); Coh. Est = correlation between cohort-specific mean L ∞ and k estimated by ADMB (individual random effects set to 0); Ind.Real = correlation between simulated L ∞ and k at the individual level; Ind.Sim = correlation between L ∞ and k at the individual level estimated by ADMB-RE. (PDF) [file pcbi.1003828.s006.pdf]

Table S3. Pearson's correlation between realized (i.e. not from the Hessian matrix) estimates of cohort-specific and of individual L_∞ (mm) and *k* (y^{-1}). We carried out 30 reproducible replicates. Coh.Sim = correlation between simulated cohort-specific mean L_∞ and *k* (individual random effects set to 0); Coh. Est = correlation between cohort-specific mean L_∞ and *k* estimated by ADMB (individual random effects set to 0); Ind.Real = correlation between simulated L_∞ and *k* at the individual level; Ind.Sim = correlation between L_∞ and *k* at the individual level estimated by ADMB-RE.

Coh.Sim	Coh.Est	Ind.Sim	Ind.Est
-0.13	-0.12	0.20	0.20
0.19	0.20	0.34	0.35
-0.73	-0.74	0.06	0.07
0.09	0.06	-0.46	-0.44
0.43	0.44	0.29	0.30
-0.14	-0.16	-0.69	-0.69
0.19	-0.24	-0.35	0.02
-0.17	-0.13	0.25	0.26
0.79	0.77	0.72	0.73
-0.81	-0.84	0.57	0.59
0.71	0.70	-0.98	-0.97
0.60	0.54	-0.23	-0.22
-0.24	-0.35	-0.46	-0.46
-0.56	-0.52	0.32	0.33
-0.98	-0.98	-0.82	-0.79
0.54	0.55	0.60	0.60
-0.55	-0.50	0.08	0.09
0.60	0.63	0.63	0.63
-0.93	-0.91	-0.70	-0.70
0.13	0.15	-0.48	-0.47
-0.16	-0.15	-0.05	-0.03
-0.92	-0.90	0.41	0.43
0.69	0.67	-0.59	-0.57
-0.54	-0.57	-0.77	-0.76

-0.68	-0.64	0.26	0.25
0.35	0.36	-0.95	-0.94
-0.11	-0.12	-0.67	-0.65
0.32	0.28	0.26	0.26
0.10	0.05	-0.66	-0.65
-0.35	-0.30	-0.47	-0.45
