# Supplementary material for: Determining Individual Variation in Growth and Its Implication for Life-History and Population Processes Using the Empirical Bayes Method
Source: PLoS Comput Biol. 2014 Sep 11;10(9):e1003828. doi: 10.1371/journal.pcbi.1003828 (PMC4161297; doi:10.1371/journal.pcbi.1003828)
Supplement: Text S1 — Processes leading to negative correlation between L∞ and k and how size-dependent survival generates a negative correlation between random effects. (PDF) [file pcbi.1003828.s007.pdf]

Text S1. Processes leading to negative correlation between L_∞ and *k* and how size-dependent survival generates a negative correlation between random effects

In marble trout, a negative correlation between L_∞ and k at the individual level (i.e. growth trajectories often crossing) may theoretically emerge from the interaction between (i) size-dependent sexual maturity, (ii) high reproductive investment, and (iii) iteroparity. Fish growing faster may reach sexual maturity in November-December at a younger age than slow growers, who may reach a bigger size at maturity the following year(s), after the May-to-September growth season(s). When the high reproductive investment causes a drastic reduction in somatic growth after sexual maturity (i.e. energy is allocated to future reproduction), fast growers/early reproducers may reach a lower adult size than slow growers/late reproducers, thus leading to size ranks that may be not maintained over time. However, a smaller reproductive investment that still allows the allocation of energy to growth after sexual maturity may lead to the emergence of a positive correlation or no correlation between L_∞ and k at the individual level. If (i-iii) are true, the plateau in body growth after sexual maturity should emerge more clearly for females, since the energetic investment in gonads relative to soma is much higher in females than in males. Although we presently do not have information on either sex of marble trout or age at spawning for individual females, the presence of both a strong positive correlation between L_∞ and k (see main text) and iteroparity in marble trout seems to exclude that reproductive investment drastically limits later growth .

We show now how the correlation between k and L_∞ can be generated by size-dependent mortality. Mortality in fishes tends to scale inversely with length [1], i.e. $\mu \approx \mu_0 + \frac{\mu_1}{L(t)}$. Such size dependent mortality results in selection on size and hence on the parameters governing growth, specifically, survival to time t, $S(t)$, of individuals with growth parameters k and L_∞ is given by:

$$\ln[S(t)] = -\int_0^t \mu_0 + \frac{\mu_1}{L(s)} ds \tag{S6.1}$$

Substituting Eq. 5 in the main text in Eq. S6.1, we have:

$$\ln[S(t)] = -\mu_0 t - \mu_1 \int_0^t \frac{1}{L_0 e^{-ks} + L_\infty(1 - e^{-ks})} ds \tag{S6.2}$$

Changing variables from t to $u = L_0 + L_\infty(e^{kt} - 1)$ and integrating gives:

$$\ln[S(t)] = -\mu_0 t - \frac{\mu_1}{kL_\infty} \ln\left[\frac{L_0 + L_\infty(e^{kt} - 1)}{L_0}\right] \tag{S6.3}$$

Thus the distribution among individuals surviving to time t is:

$$p(k, L_\infty, t) = cp(k, L_\infty, 0)\left[\frac{L_0}{L_0 + L_\infty(e^{kt} - 1)}\right]^{\frac{\mu_1}{kL_\infty}} \quad \text{(S6.4)}$$

Where c is the normalizing constant. To illustrate the effect that size-dependent survival can have on the distribution of growth parameters, we applied this model to the case where the initial distribution of k and L_∞ is the product of independent normal distributions (figure text S1). There are two important conclusions from this analysis. The first is that the selection induced-correlation between k and L_∞ is always negative. The second is that it only occurs when for small initial sizes and relatively small values of k and L_∞. For the values of k and L_∞ observed in this study, the negative correlation induced by selection is negligible, but in any case it is never positive.

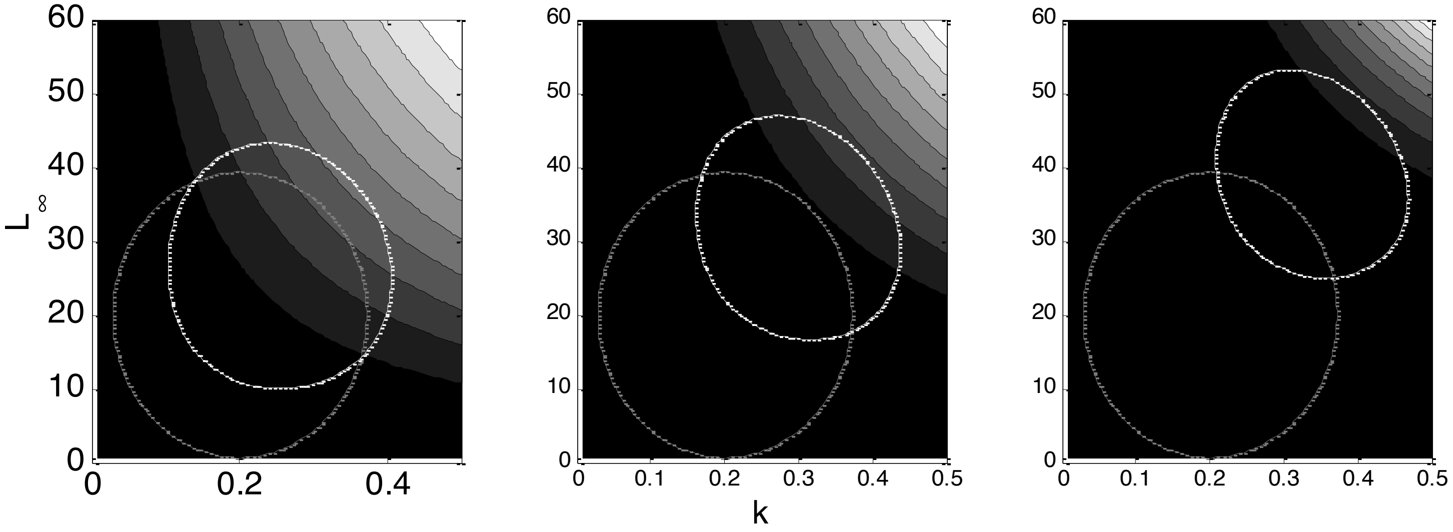

Figure text S1. Selection induced correlation among k and L_∞. In each panel, the gray ellipse indicates the initial distribution and the white indicates the distribution after selection. Both are contours where the density is 10% of its maximum value. From left to right, t = 0.01, 0.1, and 10. The remaining parameters are L_0=0.01, μ_1=10. The initial distribution for k is N(0.2, 0.0064) (y^{-1}) and for L_∞ is N(20,81) (mm). Note that these parameters were chosen to emphasize the induced correlation. For larger values of k and L_∞ the effect of selection is primarily to shift the means and variances, with little effect on covariance.

References

1. Lorenzen K. EK (2002) Density-dependent growth as a key mechanism in the regulation of fish populations: Evidence from among-population comparisons. Proc R Soc B Biol Sci 269: 49–54.
