# Supplementary material for: Determining Individual Variation in Growth and Its Implication for Life-History and Population Processes Using the Empirical Bayes Method
Source: PLoS Comput Biol. 2014 Sep 11;10(9):e1003828. doi: 10.1371/journal.pcbi.1003828 (PMC4161297; doi:10.1371/journal.pcbi.1003828)
Supplement: Text S2 — Comparison with JAGS and nlme function in R. (PDF) [file pcbi.1003828.s008.pdf]

Text S2. Comparison with JAGS and *nlme* function in R

We tried fitting the model presented in Eq. 7 in the manuscript using JAGS (see file “vb.jags.on.r” in the online repository).

However, even by using simulated data and only one parameter as a linear combination of a population-level parameter and individual random effects, we were not able to obtain model convergence. Although demonstrating that a method works is easier than demonstrating that a method does not work, we believe that it is unlikely to obtain reasonable parameter estimates using the model in Eq. 7 with JAGS. We also tested whether the *nlme* function in the nlme package of R was able to fit our random-effects model. As we show in the file “VBGF nlme.r” in the online repository, we were able to obtain convergence with simulated data. However, when using empirical data (i.e. data from Zakosjka and Gacnik) we were not able to obtain model convergence using the *nlme* function.At

http://dx.doi.org/10.6084/m9.figshare.831432 we report the JAGS and *nlme* models and code we used to try to fit the model.
