# Supplementary material for: Determining Individual Variation in Growth and Its Implication for Life-History and Population Processes Using the Empirical Bayes Method
Source: PLoS Comput Biol. 2014 Sep 11;10(9):e1003828. doi: 10.1371/journal.pcbi.1003828 (PMC4161297; doi:10.1371/journal.pcbi.1003828)
Supplement: Text S3 — Repeatability analysis. (PDF) [file pcbi.1003828.s009.pdf]

Text S3. Repeatability analysis

Repeatability measures the proportion of total phenotypic variance that can be attributed to among-individual variation, whether genetically- or environmentally-induced. High repeatability of body size means that size ranks are maintained throughout fish lifetime. Thus, to provide additional biological interpretation of the correlation between L_∞ and k at the individual level, we estimated repeatability of body size in the populations of Gacnik and Zakojska.

With longitudinal data, repeatability can be estimated as the ratio of among-individual variance over phenotypic variance [1]. We tested for potential inflation of repeatability due to thinning of populations at older ages by also estimating repeatability for fish with ages up to 3 years. We estimated repeatability using a Generalized Linear Mixed Model as implemented in the R package MCMCglmm [1,2] with age as fixed effect (12 levels for Gacnik and 9 for Zakojska for the complete data set, 3 levels for each populations when using fish with ages up to 3 years) and fish ID as random effect.

Results

We obtained very high estimates of repeatability of body size for both populations. In Gacnik the mean and 95% credible intervals were 0.75 [0.73-0.76] for all fish and 0.76 [0.75-0.78] for fish up to three years old. The

corresponding values for Zakojska were 0.66 [0.62-0.70] for all fish and 0.71 [0.66-0.74] for fish up to three years old.

References

1. Wilson AJ, Réale D, Clements MN, Morrissey MM, Postma E, et al. (2010) An ecologist's guide to the animal model. J Anim Ecol 79: 13–26.

2. Hadfield JD (2010) MCMC Methods for Multi-Response Generalized Linear Mixed Models: The MCMCglmm R Package. J Stat Softw 33: 1–22.
