# Supplementary material for: Determining Individual Variation in Growth and Its Implication for Life-History and Population Processes Using the Empirical Bayes Method
Source: PLoS Comput Biol. 2014 Sep 11;10(9):e1003828. doi: 10.1371/journal.pcbi.1003828 (PMC4161297; doi:10.1371/journal.pcbi.1003828)
Supplement: Text S4 — The Empirical Bayes algorithm. (PDF) [file pcbi.1003828.s010.pdf]

Text S4. The Empirical Bayes algorithm

Given the model specification in Eqs. 7 – 9 in the main text, the software ADMB-RE automatically carries out the Empirical Bayes (EB) algorithm. In this section, the details of the EB procedure are given with sufficient mathematical details to allow reimplementation in another programming language/package.

The likelihood given in Eq. 9 in the main text is implicitly a joint likelihood in parameters ($\boldsymbol{\theta}$) and random effects (u's and v's), although this is not explicit in our notation. The distributional assumptions $u_{ij} \sim N(0,1)$ and $v_{ij} \sim N(0,1)$ act as "penalties". The penalized likelihood (earlier referred to as the joint probability density) involved in Eq. 2 in the main text becomes

$$f(\text{data}_{ij}, u_{ij}, v_{ij}; \boldsymbol{\theta}) = \frac{1}{\sqrt{2\pi}} \exp\left(-\frac{u_{ij}^{2}}{2}\right) \frac{1}{\sqrt{2\pi}} \exp\left(-\frac{v_{ij}^{2}}{2}\right) \times \prod_{l=1}^{m_{ij}} \frac{1}{\sqrt{2\pi}\sigma_{\varepsilon}} \exp\left(-\frac{\left(L_{ijl} - L(t_{ijl}; L_{\infty}^{(ij)}(u_{ij}), k^{(ij)}(v_{ij}), t_0)\right)^2}{2\sigma_{\varepsilon}^{2}}\right) \quad \text{(S4.1)}$$

where the dependence of $L_{\infty}^{(ij)}$ and $k^{(ij)}$ on the random effects have been made explicit.

The EB parameter estimation procedure simply amount to maximizing the likelihood function $L(\boldsymbol{\theta})$ given in Eq. 2 in the main text for the above choice of f. However, this ignores the issue of how the integral in Eq. 2 in the main

text is evaluated. Because $L(\boldsymbol{\theta})$ is being evaluated repeatedly as a part of the numerical optimization algorithm used to find the maximum likelihood estimate of $\boldsymbol{\theta}$, the efficient evaluation of the integral in Eq. 2 in the main text is a critical issue. In ADMB-RE this is done via the Laplace approximation leading to an EB algorithm that iterates between the following two steps:

1. For fixed $\boldsymbol{\theta}$ maximize $f(\text{data}_{ij}, u_{ij}, v_{ij}; \boldsymbol{\theta})$ with respect to u_{ij} and v_{ij} to obtain estimates $\hat{u}_{ij}$ and $\hat{v}_{ij}$. This is done for all (i, j).
2. Update $\boldsymbol{\theta}$ by picking a value that increases the Laplace approximation of the Eq. 2 in the main text, i.e.

$$L(\boldsymbol{\theta}) = \prod_{i,j} (2\pi) \left| \det\left(H_{ij}\right) \right|^{-1/2} f(\text{data}_{ij}, \hat{u}_{ij}, \hat{v}_{ij}; \boldsymbol{\theta}). \qquad \text{(S4.2)}$$

Here, H_{ij} is the 2×2 Hessian matrix consisting of the second order partial derivatives of $\log f(\text{data}_{ij}, u_{ij}, v_{ij}; \boldsymbol{\theta})$ with respect to u_{ij} and v_{ij}, and $\det(H)$ denotes the determinant of the matrix H. The details about the "updating" (step 2 above) depends on which optimization algorithm (such as quasi-Newton) is used, and is not discussed further here.
